# Mitochondrial-Targeted Triphenylphosphonium-Conjugated Ionophores with Enhanced Cytotoxicity in Cancer Cells

**DOI:** 10.3390/molecules30224413

**Published:** 2025-11-14

**Authors:** Michał Sulik, Marta Jędrzejczyk, Magdalena Mielczarek-Puta, Jakub Hoser, Piotr Bednarczyk, Marta Struga, Adam Huczyński

**Affiliations:** 1 Department of Medical Chemistry, Faculty of Chemistry, Adam Mickiewicz University, Uniwersytetu Poznańskiego 8, 61-614 Poznań, Poland; 2 Chair and Department of Biochemistry, Faculty of Medicine, Medical University of Warsaw, Banacha 1, 02-097 Warsaw, Poland; 3 Department of Physics and Biophysics, Institute of Biology, Warsaw University of Life Sciences SGGW, Nowoursynowska 159, 02-776 Warsaw, Poland

**Keywords:** ionophore antibiotics, salinomycin, monensin, mitochondria-targeting conjugates, antiproliferative activity, triphenylphosphonium cation

## Abstract

Salinomycin and monensin represent a class of natural ionophore antibiotics with strong anticancer properties. In this paper we report on chemical modification of these compounds by conjugation with phosphonium cations for targeting conjugates to the mitochondria of cancer cells. Our findings indicate that this approach yields conjugates with enhanced anticancer activity and selectivity, outperforming not only the parent compounds but also the widely used chemotherapeutic agent, doxorubicin. Comprehensive biological and biophysical analyses proved that the conjugates target the mitochondria in cancer cells, with some of the derivatives additionally promoting generation of mitochondrial reactive oxygen species (mtROS). This targeted strategy holds significant promise for the development of effective mitochondrial-targeted novel anticancer agent.

## 1. Introduction

Among various organelles present in eukaryotic cells that contribute to the maintenance of cellular homeostasis, mitochondria play a pivotal role [1]. These organelles are involved in numerous essential biochemical pathways; however, due to their fundamental function in cellular respiration and ATP production through oxidative phosphorylation, they are often called the “powerhouses” of the cell [1]. Moreover, recent studies have suggested that mitochondria are closely associated with intrinsic and extrinsic apoptotic pathways [2,3,4]. This makes them highly attractive molecular targets for drugs used in the treatment of various diseases, including cancer, as mitochondrial dysfunction has been implicated in both tumorigenesis and tumor progression [2,3,4]. Notably, according to the World Health Organization (WHO) forecasts, the global incidence of cancer is expected to rise rapidly in the coming years, reaching as many as 30 million new cases in 2040 [5]. This underscores the urgent need for development of novel effective anticancer drugs. One promising strategy involves the design of compounds that selectively target the mitochondria of cancer cells. It is possible due to a significantly more negative mitochondrial membrane potential in cancer cells (approximately −220 mV) compared to that in normal cells (around −160 mV), which creates a hyperpolarized state that promotes the uptake of various lipophilic organic cations [6]. Among such mitochondria targeting moieties, triphenylphosphonium cation (TPP^+^) is one of the most widely studied and utilized [3,4,7,8]. It has been established that conjugation of mitochondria-targeting moieties with anticancer agents or molecular sensors enhances their properties (cytotoxicity and sensing activity, respectively) compared to their unconjugated counterparts [9]. This finding suggests that the conjugation of diverse chemical compounds with TPP+ represents a promising research strategy, as the resulting hybrids can be selectively directed to the mitochondria of cancer cells. In this context, it is worth highlighting a class of naturally derived compounds with high potential in targeting malignant cells, i.e., ionophore antibiotics.

**Figure 1:**
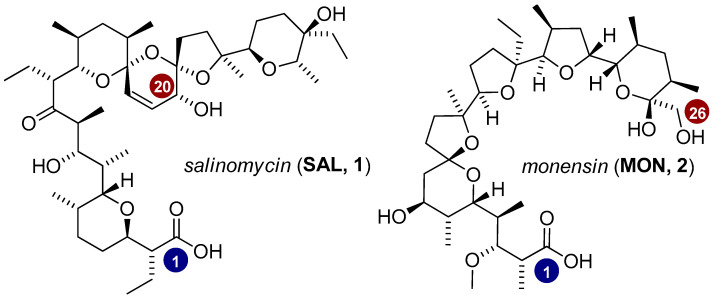
Chemical structures of the two main representatives of ionophore antibiotics—salinomycin and monensin.

Ionophore antibiotics are isolated from bacterial stains of the Streptomyces genus and are commonly used in veterinary medicine [10,11]. Their ability to coordinate metal cations and transport them across biological membranes underlies their broad spectrum of biological properties, including antibacterial, antiparasitic, and antifungal activities [10,11]. Two of the major representatives of ionophore antibiotics—salinomycin and monensin—have also gained considerable attention as potential anticancer agents, owing to their high activity confirmed in numerous studies. The exceptionally high anticancer activity of salinomycin (SAL, Figure 1) was first reported in 2009 when high-throughput screening of 16,000 biologically active compounds identified SAL as the most effective agent in eliminating breast cancer stem cells [12]. This ionophore antibiotic has demonstrated tumor growth inhibition in vivo with approximately 100-fold greater selectivity than paclitaxel [12]. SAL has also been shown to suppress cell migration and proliferation, as well as to induce apoptosis and autophagy [13,14,15,16,17]. In addition to its ability to transport cations across biological membranes, the proposed mechanisms underlying its anticancer effects include inhibition of the Wnt and Hedgehog signaling pathways [18,19,20], disruption of multidrug efflux systems [21,22], generation of reactive oxygen species (ROS) [23,24], and induction of DNA damage [25]. The high anticancer efficacy of another ionophore antibiotic, monensin (MON, Figure 1), was also identified in screening studies involving 5000 known drugs and drug-like compounds [26]. The results indicated that four compounds, including MON, selectively inhibited the growth of prostate cancer cells at nanomolar concentrations, while exerting no significant effects on the proliferation of normal prostate epithelial cells [26].

In addition, MON has been shown to suppress cell proliferation in various other cancer types, such as abnormal colon cells, lymphoma, and myeloma, primarily by inducing cell cycle arrest and disrupting mitochondrial transmembrane potential [27,28,29]. These findings highlight the exceptionally potent anticancer activity of both ionophore antibiotics. A rational design strategy involving their conjugation with TPP^+^ may lead to the development of highly bioactive hybrid compounds capable of selectively targeting the mitochondria of cancer cells. Our previous research led to the development of a series of SAL and MON hybrids conjugated with TPP+ and other phosphonium cations via an ester linker at position C1. These compounds demonstrated enhanced antiproliferative activity and selectivity across various cancer cell lines, compared to those of the parent ionophore antibiotics [30,31]. Moreover, both biological and biophysical studies have confirmed that these compounds are directed toward the mitochondria of cancer cells, ultimately leading to apoptosis [30,31]. It is important to note that position C1 is essential for preserving the ionophoretic properties of SAL and MON. Consequently, an interesting direction for further research involves the conjugation of ionophore antibiotics with TPP^+^ via alternative positions. One such conjugate—SAL modified with TPP^+^ at position C20—has already been synthesized in our laboratory. Physicochemical analyses have demonstrated that this compound retains mitochondria-targeting capability and facilitates K^+^/H^+^ exchange across both artificial and biological membranes [32]. The aim of the present study was to synthesize SAL and MON derivatives conjugated with TPP^+^, while preserving the free C1 carboxylic group. These compounds were subsequently subjected to comprehensive biological and biophysical evaluation, with particular emphasis on their ability to selectively target the mitochondria of cancer cells.

## 2. Results and Discussion

### 2.1. Analogs Design and Synthesis

Besides the carboxyl group, widely explored issues in the study of ionophore antibiotics are their hydroxyl functionalities. In this context, the hydroxyl group at position C20 in the structure of SAL and the hydroxyl group at position C26 in the structure of MON are considered the most reactive (Figure 1). Numerous direct transformations of these functional groups into derivatives such as esters, carbonates or *O*-urethanes have been reported [33,34,35,36]. Moreover, the modification of hydroxyl groups into other functionalities—such as azide or amine [32,37]—represents a valuable synthetic strategy, enabling the expansion of known derivatives to include, for instance, amides, ureas, or triazole-based compounds [37,38,39,40,41,42].

As demonstrated in previous studies, the chemical nature of the linker connecting the ionophore to the TPP^+^ moiety plays a crucial role in the design of such conjugates [30,31]. Factors such as the linker stability and alkyl chain length can significantly influence the biological activity of the resulting analogs. Therefore, in the present study, we selected 1,2,3-triazole, ester, and amide linkers in order to systematically investigate the relationship between the chemical characteristics of the linker and the biological and biophysical properties of the resulting hybrid molecules. In addition to TPP^+^-based conjugates, a bioconjugate incorporating tributylphosphine was also synthesized to determine whether the aromatic nature of the substituent is essential for maintaining the hybrid’s activity. Furthermore, doubly modified derivatives of SAL and MON were prepared, in which, besides modifications at positions C20 and C26, respectively, additional structural modifications were introduced at position C1.

**Scheme 1:**
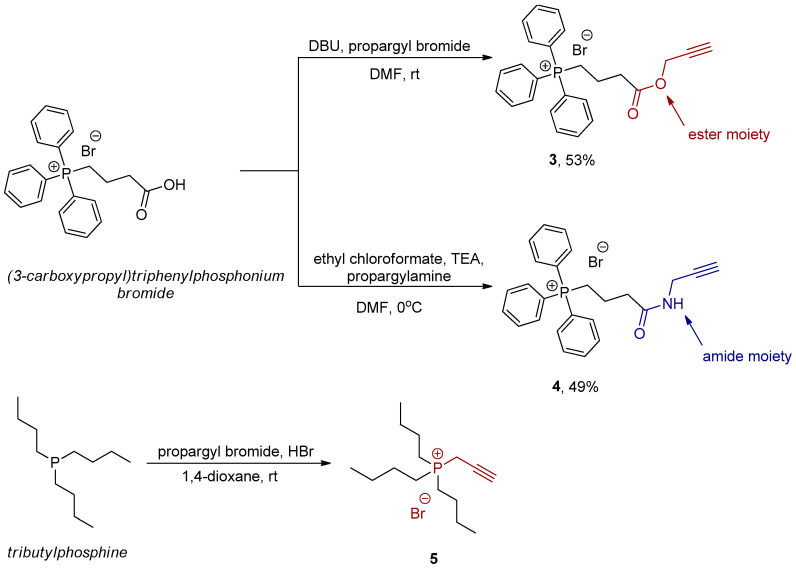
Synthesis of propargyl phosphonium salts as precursors for click reaction.

The method most commonly employed for the synthesis of 1,2,3-triazole rings is the Huisgen 1,3-dipolar cycloaddition, often referred to as “click chemistry.” This reaction utilizes organic azides and alkynes as substrates. Consequently, it was necessary to obtain suitable precursors for subsequent bioconjugation reactions. Tributylphosphine and (3-carboxypropyl)triphenylphosphonium bromide were converted into their respective propargyl derivatives (Scheme 1). Notably, the carboxylic acid functional group was transformed into the corresponding propargyl ester and amide (compounds **3** and **4**), with reaction yields of approximately 50%. Tributylphosphine was converted into a propargyl phosphonium salt (compound **5**); however, the product was not isolated but instead used in crude form for subsequent transformations (Scheme 1).

**Scheme 2:**
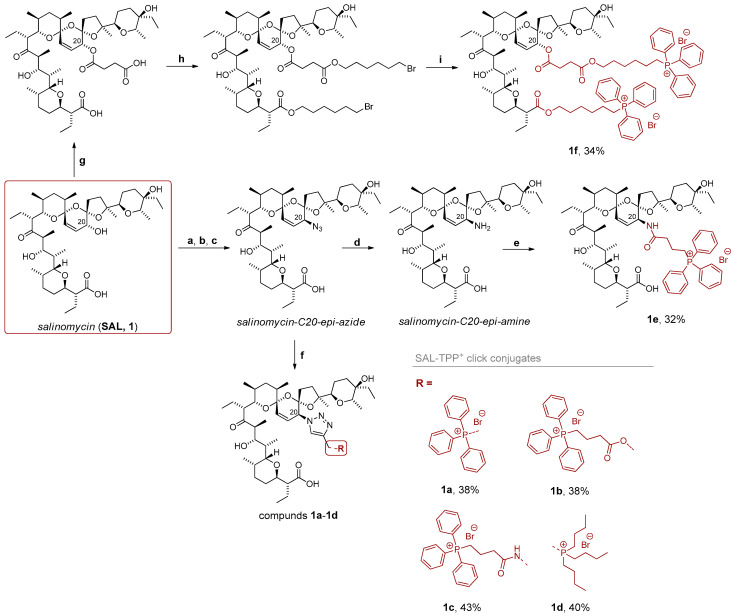
Synthesis of phosphonium salts of SAL. Reagents and conditions: (**a**) TMS(CH_2_)_2_OH, DMAP, TCFH, CH_2_Cl_2_, 0 °C to rt, 24 h; (**b**) PPh_3_, DIAD, DPPA, THF, 0 °C to rt, 24 h; (**c**) TBAF, THF, rt, 24 h; (**d**) PPh_3_, H_2_O, THF, rt, 24 h; (**e**) (3-carboxypropyl)triphenylphosphonium bromide, TEA, DMF, 0 °C to rt, 24 h; (**f**) triphenylpropargylphosphonium bromide, **3**, **4** or **5**, DIPEA, CuI, CH_3_CN, rt, 24 h; (**g**) succinic anhydride, pyridine, 60 °C, 24 h; (**h**) DBU, 1,6-dibromohexane, toluene, reflux, 24 h; (**i**) PPh_3_, CH_3_CN, reflux, 72 h.

Introduction of the azido functionality at position C20 of SAL involves a three-step synthetic sequence comprising: (i) protection of the C1 hydroxyl group using a silyl alcohol, (ii) a Mitsunobu reaction employing diphenylphosphoryl azide (DPPA) as the azide source, and (iii) deprotection of position C1 using TBAF (Scheme 2). Notably, this sequence results in an inversion of configuration at the asymmetric C20 carbon center. The resulting azide serves as a convenient substrate for the click reaction.

In the context of SAL derivatives, the most suitable approach to the click reaction is via the Meldal method, which utilizes CuI as a source of copper(I) ions. This strategy enabled the synthesis of triazole-linked SAL conjugates with triphenylpropargylphosphonium bromide and propargylated precursors **3**, **4**, or **5** (compounds **1a**–**1d**), with reaction yields ranging from 38% to 43% (Scheme 2). In the subsequent step, the C20 azide of SAL was reduced to the corresponding amine precursor via the Staudinger reaction (Scheme 2). Given the availability of the carboxylic phosphonium salt previously used for the synthesis of propargyl precursors, it was possible to directly obtain the corresponding amide conjugate (compound **1e**, Scheme 2) with the yield of 32%. Furthermore, a disubstituted SAL–TPP^+^ conjugate was targeted. To this end, an additional carboxylic acid moiety was introduced into the SAL scaffold via reaction with succinic anhydride (Scheme 2). Since both carboxylic acid groups exhibited similar reactivity, subsequent esterification with 1,6-dibromohexane followed by nucleophilic substitution with triphenylphosphine afforded the final disubstituted conjugate (compound **1f**) in 34% yield (Scheme 2).

**Scheme 3:**
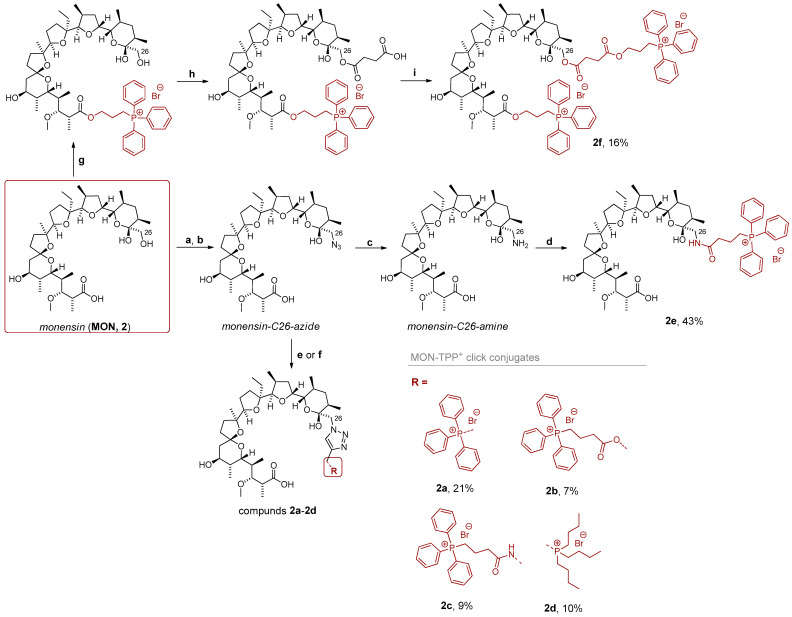
Synthesis of phosphonium salts of MON. Reagents and conditions: (**a**) TsCl, Na_2_CO_3(aq)_, toluene, rt, 72 h; (**b**) NaN_3_, DMSO, rt, 72 h; (**c**) H_2_, Pd/C, MeOH, rt, 72 h; (**d**) (3-carboxypropyl)triphenylphosphonium bromide, ethyl chloroformate, TEA, DMF, −5 °C to rt, 24 h; (**e**) triphenylpropargylphosphonium bromide or **4**, DIPEA, CuI, CH_3_CN, rt, 24 h; (**f**) **3** or **5**, sodium ascorbate, CuSO_4_, H_2_O/MeOH, rt, 24 h; (**g**) DBU, (3-carboxypropyl)triphenylphosphonium bromide, toluene, reflux, 24 h; (**h**) succinic anhydride, pyridine, 60 °C, 24 h; (**i**) DBU, (3-carboxypropyl)triphenylphosphonium bromide, toluene, reflux, 24 h.

A similar strategy was employed for the synthesis of new MON derivatives; however, the synthetic procedures required certain modifications. The synthesis of the C26-azide derivative of MON was carried out in a two-step sequence involving tosylation of the hydroxyl group followed by a nucleophilic substitution with sodium azide (Scheme 3). Subsequently, a click reaction was performed with the aforementioned propargyl precursors. Interestingly, the Meldal protocol proved effective only for the synthesis of compounds **2a** and **2c**. In contrast, the formation of conjugates **2b** and **2d** required the application of Sharpless conditions, in which Cu(I) ions were generated in situ via the reaction of sodium ascorbate with CuSO_4_ (Scheme 3). The C26-azide group was then reduced to a primary amine using hydrogen as the reducing agent (Scheme 3). The availability of the C26-amino functionality enabled the synthesis of an amide-linked MON–TPP^+^ conjugate **2e**. This procedure is well-established and was previously reported by our group [39].

To obtain a doubly substituted MON–TPP^+^ conjugate, several synthetic approaches were evaluated. The most effective route involved the use of 3-bromopropyltriphenylphosphonium bromide. In the first step, a C1-ester of MON was prepared via the reaction with this precursor in the presence of DBU (Scheme 3). Subsequently, an additional carboxylic acid group was introduced through the reaction with succinic anhydride, followed by a second esterification step (Scheme 3). Compound **2f** was obtained with a yield of 16%.

The purities and structural integrities of the newly synthesized derivatives (**1a**–**1f** and **2a**–**2f**) were confirmed using spectroscopic techniques, including ^1^H, and ^13^C nuclear magnetic resonance (NMR) spectroscopy, as well as high-resolution mass spectrometry (HRMS). The most characteristic signals in the ^13^C NMR spectra of SAL derivatives can be attributed to the ketone group (213.3–215.8 ppm) and the carboxyl group at position C1 (170.9–173.3 ppm). In the ^13^C NMR spectra of MON derivatives, the most diagnostically relevant signal was also attributed to the carboxyl functionality located at position C1. This resonance consistently appeared within a narrow chemical shift range of 171.6–176.6 ppm, with its exact position depending on the nature of the compound. In the ^1^H NMR spectra of “click”-type MON derivatives, the most characteristic signal is a singlet corresponding to the proton in the triazole ring. However, in most compounds, this signal overlaps with those assigned to the aromatic protons from the triphenylphosphonium substituent. An exception is compound **2d**, which bears an aliphatic substituent; in this case, a distinct singlet is observed at 7.90 ppm. HRMS analysis corroborated the formation of the target compounds, with the molecular ion peak [M]^+^, [M]^2+^, or [M+Na]^2+^ observed as the main one (100% relative intensity).

### 2.2. Biological Activity

#### 2.2.1. Cytotoxic Activity Studies

A comprehensive biological investigation was undertaken to assess the cytotoxic properties of SAL, MON and their derivatives (**1a**–**1f** and **2a**–**2f**), as well as their potential antiproliferative efficacy across a panel of eight selected cell lines. Cell viability was evaluated using the MTT colorimetric assay on six human cancer cell lines (SW480, SW620, PC3, MDA-MB-231, A549, and MiaPaCa) and two non-malignant cell lines (HaCaT and V79). The antiproliferative effects were quantified by determining the half-maximal inhibitory concentration (IC50). Additionally, the selectivity index (SI), defined as the ratio of cytotoxicity in non-cancerous to cancerous cells, was calculated to evaluate the compounds’ specificity toward malignant cells. Doxorubicin served as the reference drug in this study.

The impact of bioconjugation was not uniform and varied depending on the specific derivatives examined. In the case of SAL, the resulting conjugates retained antiproliferative activity within the micromolar range; however, their potency was generally lower compared to that of the unmodified ionophore. Among the “click”-type conjugates, compound **1d** exhibited the highest activity, despite lacking aromatic rings in its structure (Table 1). Within this group, compound **1a** also showed notable activity against certain cancer cell lines and was the only triazole derivative to surpass the biological activity of SAL (IC_50,**1a**_ = 1.2 µM vs. IC_50,SAL_ = 2.7 µM against the PC3 cell line, Table 1). The most potent conjugate among all SAL derivatives was compound **1f**, which is a doubly modified derivative. It demonstrated superior antiproliferative activity compared to SAL across most of the tested cancer cell lines, with IC_50_ values ranging from 0.3 to 1.7 µM (Table 1). When comparing these data with previously reported IC_50_ values for ester derivatives at position C1 of SAL [31], it can be concluded that, from the medicinal chemistry perspective, conjugation of the SAL molecule with the TPP^+^ moiety at the C1 carboxyl group is more favorable than modification at the C20 hydroxyl group.

In the case of MON, a different pattern was observed, as the majority of the synthesized conjugates exhibited enhanced antiproliferative activity compared to that of the unmodified ionophore. The exceptions were the amide derivatives **2c** and **2e**, with compound **2e** showing an almost complete loss of biological activity (Table 1). Among all synthesized derivatives, of both MON and SAL, the highest activity was demonstrated by compound **2a**, a “click”-type derivative, with IC_50_ values ranging from 0.3 to 0.7 µM (Table 1). Also noteworthy is compound **2f**, a doubly modified MON derivative, which consistently outperformed the parent ionophore in terms of antiproliferative activity across all tested cancer cell lines (Table 1). When comparing these results with previously reported IC_50_ values for C1-substituted MON derivatives [30], it becomes evident that, in contrast to SAL, the bioconjugation of MON with the TPP^+^ moiety can be effectively performed at both positions C1 and C26 to yield highly bioactive conjugates.

In the context of developing novel anticancer agents, the impact of the synthesized compounds on healthy cells is a critical factor. It was observed that, in the vast majority of cases, the human non-cancerous HaCaT cell line exhibited greater sensitivity to the ionophores and their derivatives compared to the animal-derived V79 cell line (Table 1). Notably, all synthesized SAL derivatives displayed reduced cytotoxicity toward the reference (non-cancerous) cell lines compared to that of the unmodified ionophore (Table 1). In contrast, the trend was reversed for MON, where only the biologically inactive amide derivatives **2c** and **2e** exhibited lower cytotoxicity toward HaCaT cells than that of the parent compound (Table 1).

A key parameter in assessment of the therapeutic potential of new anticancer compounds is their selectivity, defined as the ability to target cancer cells while sparing healthy cells. Accordingly, selectivity indices (SI) were calculated for all obtained compounds (Table 2). The highest SI values were observed in the V79 cell line, likely due to its greater resistance to ionophore antibiotics (Table 2). However, a notable improvement in selectivity was observed in many cases upon conjugation with the TPP^+^ moiety, particularly with respect to the HaCaT cell line. The most active SAL derivative, compound **1f**, also demonstrated the highest selectivity, with SI values of 5.7 and 4.3 against the PC3 and MiaPaCa cancer cell lines, respectively (Table 2). As mentioned earlier, MON derivatives exhibited strong antiproliferative activity, but they also showed considerable cytotoxicity toward the HaCaT cell line. As a result, their selectivity indices were generally low, although for the most active derivative (compound **2a**), SI values of 2.0 were achieved against the A549 and MiaPaCa cell lines (Table 2).

It is also essential to compare biological activities of the obtained derivatives with that of doxorubicin, used as a reference compound. Doxorubicin exhibits high anticancer activity across multiple cancer cell lines; however, some of the synthesized conjugates, in particular compounds **1f**, **2a**, and **2f**, surpassed it in terms of IC_50_ values (Table 1). A major drawback of doxorubicin in clinical applications is its pronounced cytotoxicity toward healthy cells. In contrast, all tested SAL and MON bioconjugates demonstrated lower cytotoxicity toward the reference HaCaT (with the exception of doubly modified **1f**) and V79 cell lines (except **2f**) than doxorubicin (Table 1). While the selectivity indices of doxorubicin were, in many cases, comparable to those of the tested derivatives, several of the newly synthesized analogs, particularly those highlighted above, exhibited markedly higher SI values, underscoring their potential as more selective anticancer agents (Table 2).

#### 2.2.2. Mitochondria Activity

Over the past years the understanding of mitochondrial functions in cancer biology has evolved significantly, establishing these organelles as central regulators of tumor cell metabolism, redox balance, and apoptotic signaling. The unique bioenergetic features and membrane potential of cancer cell mitochondria present a therapeutic opportunity for selective targeting, with mitochondrial-directed agents showing promise in overcoming resistance to conventional treatments [43]. In this context, the use of fluorescent dyes such as MitoTrackers has become a standard practice, providing critical insight into mitochondrial dynamics. MitoTracker Green stains mitochondria independently of membrane potential, enabling accurate assessment of mitochondrial mass, while MitoTracker Red CMXRos accumulates in a membrane potential-dependent manner, serving as a reliable indicator of mitochondrial function and integrity.

**Figure 2:**
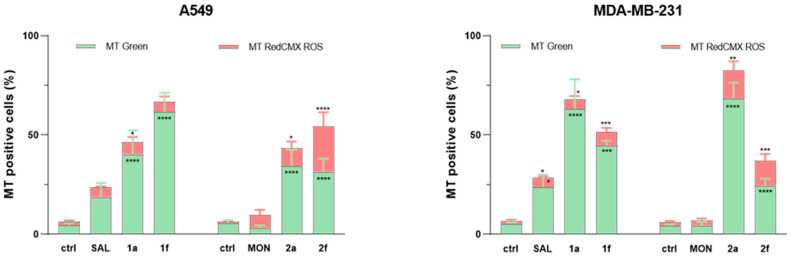
Flow cytometry analysis of MitoTracker RedCMXRos and MitoTracker GreenFM mitochondrial staining in A549 and MDA-MB-231 cells after treatment with SAL, MON and their derivatives **1a**, **1f**, **2a** and **2f** for 48 h at their IC_50_ concentrations. Untreated cells were used as the control. The bars % of MitoTrackers positive cells as compared to the control. The results are presented by means ± SD, n = 4. Statistical significance was determined at *p* < 0.05 (*); *p* < 0.01 (**); *p* < 0.001 (***) and *p* < 0.0001 (****) by 1-way ANOVA.

**Figure 3:**
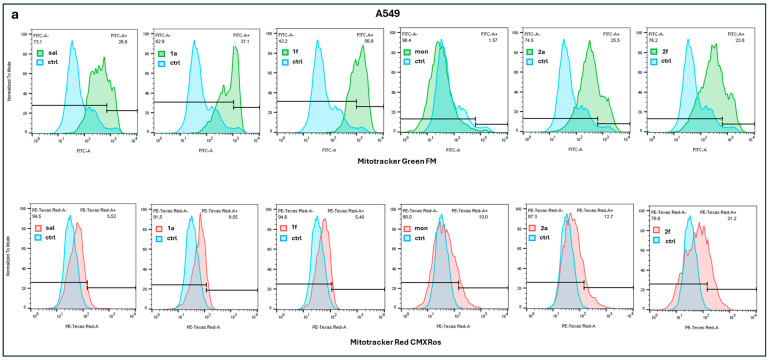

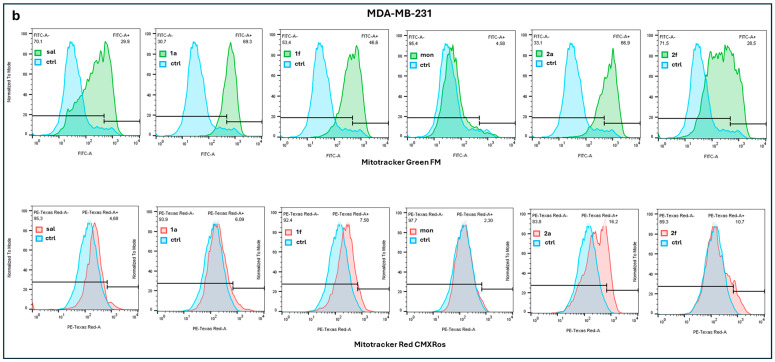
Results of a representative experiment by MitoTracker Red CMXRos and MitoTracker Green FM fluorescence assays of A549 (**a**) and MDA-MB-231 (**b**) cells after treatment with SAL, MON and their derivatives **1a**, **1f**, **2a**, **2f**, analyzed by flow cytometry. Ctrl means control.

In the present study, we evaluated SAL, MON and a panel of their structurally modified derivatives (**1a**, **1f**, **2a**, **2f**) for their potential as mitochondria-targeting agents in human cancer A549 and MDA-MB-231 cell lines. Flow cytometry-based assays revealed that all derivatives demonstrated enhanced mitochondrial mass relative to their parent compounds, as indicated by increased MitoTracker Green signal intensity. Notably, the “click”-type derivatives **1a** and **2a** exhibited the most pronounced mitochondrial mass accumulation in MDA-MB-231 cells, with 63.03% and 67.4% of cells, respectively, scoring positive for MTGreen fluorescence. Conversely, in the A549 cell line, derivative **1f**, bearing double modifications on the SAL scaffold, elicited the strongest mitochondrial signal, marking 61.6% of the population as MTGreen positive. Interestingly, in A549 cells, derivative **1a** showed comparable levels of mitochondrial targeting to **2a** and **2f**, indicating potential cell line-specific differences in uptake or mitochondrial trafficking efficiency (Figure 2 and Figure 3a,b). These findings underscore the impact of specific structural modifications on subcellular localization and suggest that mitochondrial targeting by these agents is both compound- and cell-type dependent.

In contrast, mitochondrial function and integrity estimated by MTRed CMXRos assay revealed a slight increase in red fluorescence relative to that estimated by MTGreen in all studied cases. The highest levels of MTRed CMXRos positive stained cells were observed after treatment with doubly modified MON derivatives **2f** both in A549 and MDA-MB-231 cells (22.5% and 15.5% positive cells, respectively) and after treatment with **2a** in MDA-MB-231 cells (13.4% of positive cells). Notably, **2a** and **2f** derivatives exhibited higher red fluorescence than their SAL analogs (**1a** and **1f**), especially doubly modified **2f** for which the ratio of green and red positive stained cells was close to 1:1 in A549 cells (Figure 2 and Figure 3a,b). These findings may suggest a balanced increase in the mitochondrial mass and functional integrity.

To conclude, the increase in the percentage of MitoTracker Green-positive cells, accompanied by mildly elevated levels of MitoTracker Red fluorescence in both cancer cell lines, are similar to those obtained previously [30,31] and may indicate an enhancement in mitochondrial mass without the corresponding changes in mitochondrial membrane potential (ΔΨm). This dissociation between mitochondrial content and membrane polarization suggests that the tested compounds may induce mitochondrial biogenesis or fissions independently of functional activation of the organelle. Mitochondrial fission is a dynamic cellular process in which a single mitochondrion divides into smaller organelles, what is essential for mitochondrial quality control, redistribution during cell division, and adaptation to metabolic demands. The fission typically occurs in response to cellular stress, changes in energy requirements, or during mitosis to ensure proper organelle inheritance. It is mediated by the recruitment of cytosolic GTPase dynamin-related protein 1 (Drp1) to the outer mitochondrial membrane, where it oligomerizes and constricts mitochondria to facilitate scission [43,44,45,46].

In the present study, the observed disproportion between MitoTracker Green and MitoTracker Red CMXRos fluorescence, with the MTGreen signal markedly exceeding that of MTRed, mainly for SAL and its derivatives, suggests that an increase in mitochondrial mass did not correspond to a parallel enhancement in mitochondrial membrane potential (ΔΨm). MitoTracker Green stains mitochondria independently of membrane potential and reflects mitochondrial abundance or biogenesis, whereas MTRed CMXRos selectively accumulates in the active mitochondria with intact ΔΨm. Therefore, the elevated green fluorescence alongside the moderately increased red signal may indicate that newly formed or accumulated mitochondria are structurally present but functionally impaired or less polarized.

This phenomenon is consistent with the previous reports [30,31] showing that exposure to ionophores can induce mitochondrial biogenesis or fission without necessarily improving mitochondrial activity. Such a decoupling between mitochondrial mass and function has been described as a compensatory or dysregulated response, often preceding mitophagy or apoptosis. It is also possible that mitochondrial depolarization, induced by the ion imbalance mediated by studied compounds, contributes to lower MTRed accumulation despite increased mitochondrial content. These findings highlight the importance of assessment of both mitochondrial mass and function, when evaluating mitochondrial responses to therapeutic agents, as an increase in the organelle abundance may not necessarily reflect the improved bioenergetic capacity, but rather result from a compensatory or stress-induced remodeling of the mitochondrial network. The latter phenomenon may be interpreted as a cellular self-defense response to mitotoxic stress, aimed at compensating for mitochondrial damage through increased organelle biogenesis. However, the persistence of low membrane potential in the newly formed mitochondria indicates that this defense mechanism is only partially effective, resulting in the accumulation of structurally present but functionally impaired mitochondria. Thus, ionophores do not merely disrupt the ion balance but actively reshape mitochondrial morphology, often tipping the balance toward fission in response to metabolic or oxidative stress.

#### 2.2.3. Mitochondrial ROS Production

**Figure 4:**
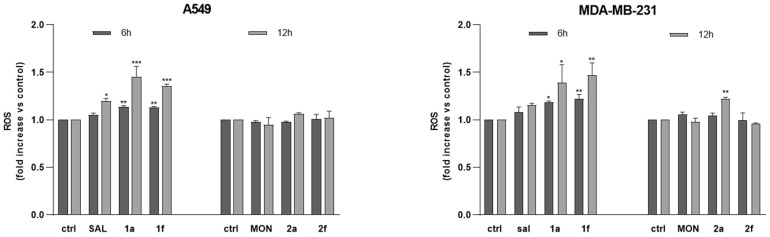
The effect of SAL, MON and their derivatives **1a**, **1f**, **2a** and **2f** on mitochondrial ROS production in A549 and MDA-MB-231 cells. The bars show the fold change in the mean fluorescent intensity as compared to the control. The cells were incubated for 6 h and 12 h with the compounds studied at their IC_50_ concentrations, labeled with MitoROS 580 dye and analyzed by microplate-based fluorimetry. Untreated cells were used as the control. The results are presented by means ± SD, n = 4. Statistical significance was determined at *p* < 0.05 (*); *p* < 0.01 (**) and *p* < 0.001 (***), by 1-way ANOVA.

Mitochondrial ROS (mtROS) generation is a well-documented consequence of disrupted mitochondrial homeostasis, often resulting from altered membrane potential and impaired electron transport chain function [47,48,49]. In the present study, mtROS levels were assessed following the 6 h and 12 h treatments with all tested compounds. Interestingly, SAL, **1a** and **1f**, but not MON and its derivatives (except for “click” type conjugate **2a**), induced mtROS production in both cancer cell lines, with a more pronounced increase observed after 12 h than 6 h of treatment. Moreover, **1a** and **1f** were more active in ROS production than SAL itself (Figure 4).

The differences in generation of mitochondrial reactive oxygen species following the treatment with SAL and MON likely reflect distinct mechanisms of ion transport and mitochondrial targeting. SAL, a potassium-selective ionophore, has been shown to induce mitochondrial dysfunction by disrupting K^+^ homeostasis and causing sustained mitochondrial membrane depolarization. This perturbation can impair electron transport chain (ETC) function, promote electron leakage particularly in complexes I and III and ultimately lead to excessive mitochondrial ROS generation [50].

In turn, MON mediates electroneutral Na^+^/H^+^ exchange and alters pH [51,52,53]. Therefore, based on the obtained results, it seems that under the same experimental conditions, its impact on ROS is minimal compared to that of the agents like SAL that directly perturb ΔΨm and cause ETC backpressure. Moreover, no ROS generation in MON-treated cells may also be due to efficient compensatory mechanisms, including enhanced upregulation of antioxidant defenses, which are insufficient to counteract the oxidative stress induced by SAL. Notably, our findings indicate that comparable cytotoxic effects of SAL, MON and their derivatives are obtained by different mechanisms. The comparison of MON and SAL derivatives has shown differences in green and red ratio fluorescence. It seems that the newly formed mitochondria after the treatment with **2a** or **2f** preserved ΔΨm, which results in the lack of electron transport chain ROS generation (except for **2a** in MDA-MB-231 cells). In contrast, the impaired ΔΨm status observed for **1a** and **1f** leads to excessive ROS and compensatory mitochondrial mass gain.

### 2.3. Biophysical Studies

#### 2.3.1. Mitochondrial Respiration Rate

**Figure 5:**
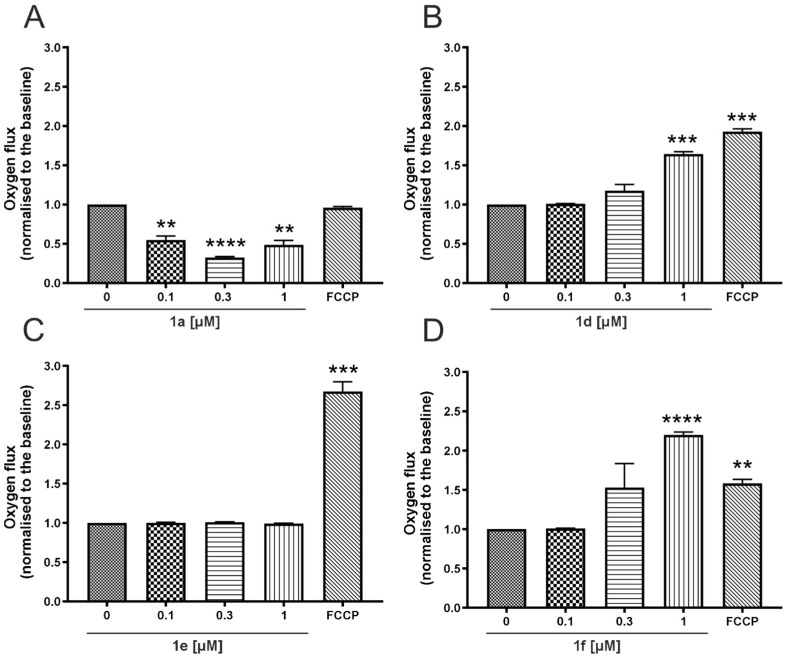
Impact of SAL derivatives on the oxygen consumption rate in whole non-perforated A549 cells cultured in starving DMEM (Dulbecco’s Minimal Essential Medium). Bars illustrate the effects of the tested compounds at different concentrations (0.1, 0.3 and 1 μM) on the oxygen consumption rate of A549 cells, measured using high-resolution respirometry. The compounds analyzed include SAL analogs: (**A**) **1a**, (**B**) **1d**, (**C**) **1e** and (**D**) **1f**; their effects are normalized to the oxygen consumption rate recorded after stabilization, prior to any compound addition. Complete mitochondrial uncoupling was induced with 2 μM FCCP. Data are presented as mean ± SD, with n = 4. Statistical significance was assessed by one-way ANOVA, with *p*-values of <0.01 (**), <0.001 (***), and *p* < 0.0001 (****) considered significant.

Changes in cellular respiration in response to the derivatives of SAL and MON were assessed using the Oxygraph-2K system. High-resolution respirometry was employed to measure oxygen consumption in live, whole non-permeabilized A549 cells, allowing evaluation of the impact of SAL and MON analogs on mitochondrial function. This approach also provided insight into the compounds’ potential to accumulate within mitochondria, as reflected by alterations in the cellular respiration rate. The baseline oxygen flux was approximately 25 pM/s·mL, and all subsequent changes were normalized to this value. To determine maximal respiratory capacity, FCCP was added during the measurements. Figure 5 shows changes in oxygen flux of HBE cells in the presence of SAL and its conjugates. Derivates **1d** and **1f** (Figure 5B,D) increased cellular respiration in 0.3 and 1 µM. In further application of FCCP **1d** uncoupled mitochondria far more while **1f** was characterized by reduction in the respiration rate, which could implicate destabilization of mitochondria. Significant decrease in oxygen flux was observed for **1a** (Figure 5A) while 1e did not cause any changes (Figure 5C).

**Figure 6:**
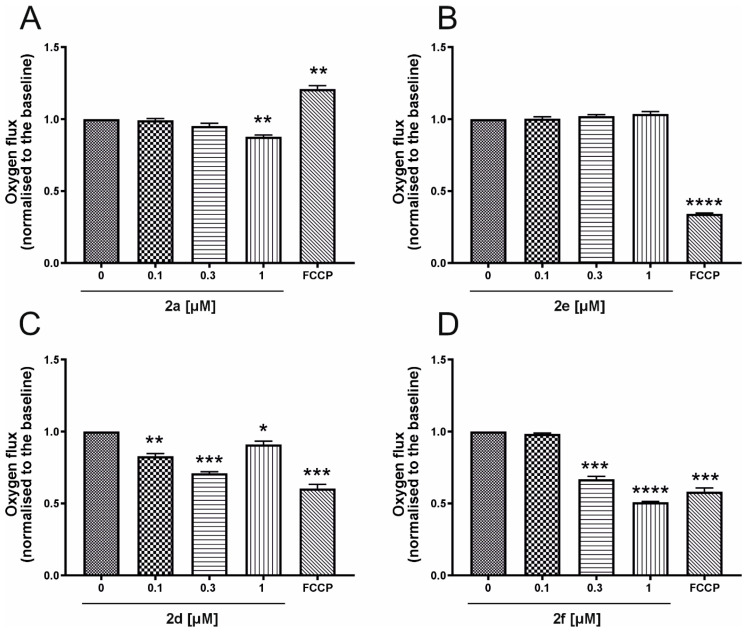
Impact of MON derivatives on the oxygen consumption rate in whole non-perforated A549 cells cultured in starving DMEM (Dulbecco’s Minimal Essential Medium). Bars illustrate the effects of the tested compounds at different concentrations (0.1, 0.3 and 1 μM) on the oxygen consumption rate of A549 cells, measured using high-resolution respirometry. The compounds analyzed include MON analogs: (**A**) **2a**, (**B**) **2d**, (**C**) **2e** and (**D**) **2f**; their effects are normalized to the oxygen consumption rate recorded after stabilization, prior to any compound addition. Complete mitochondrial uncoupling was induced with 2 μM FCCP. Data are presented as mean ± SD, with n = 4. Statistical significance was assessed by one-way ANOVA, with *p*-values of <0.05 (*), <0.01 (**) and <0.001 (***), and *p* < 0.0001 (****) considered significant.

As shown in Figure 6, several MON conjugates significantly reduced oxygen flux, with compound **2f** exhibiting the strongest effect (Figure 6A,C,D). Compound **2a** (Figure 6A) only reduced baseline respiration at the highest tested concentration (1 µM). In contrast, compound **2e** (Figure 6B) had no observable impact on oxygen consumption. Interestingly, following FCCP administration, no increase in oxygen flux was detected in the presence of MON analogs, suggesting a potential direct interaction with the mitochondrial respiratory chain. Notably, FCCP failed to stimulate respiration in all experiments involving MON derivatives.

#### 2.3.2. Interaction of Salinomycin Derivatives with Lipid Membranes

**Figure 7:**
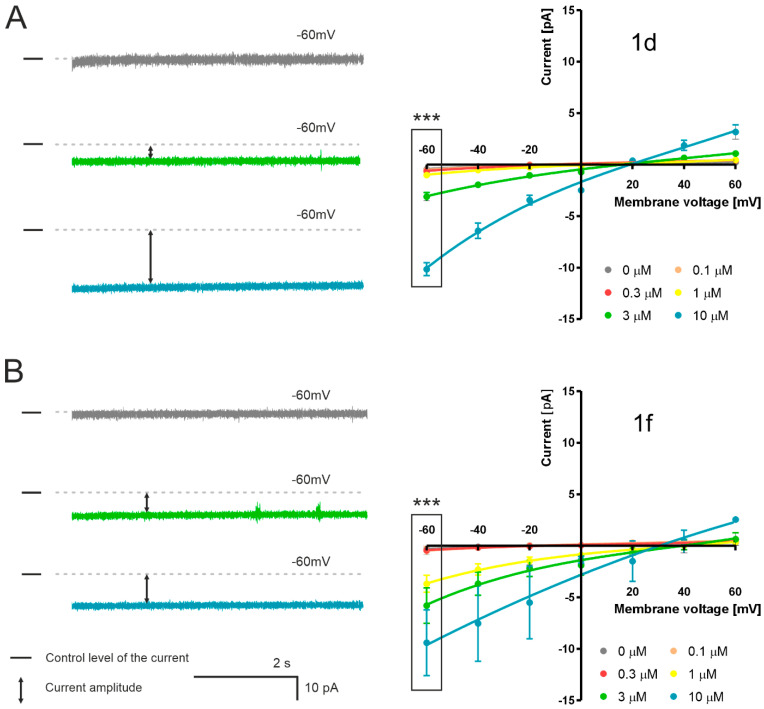
Current–time recordings and current–voltage (I–V) relationships for ionic currents across lipid bilayer membranes under a 50/150 mM KCl (cis/trans) gradient. The left panel displays representative current traces recorded at −60 mV in control conditions and in the presence of 3 or 10 μM of (**A**) compound **1d**, (**B**) compound **1f**. The right panel shows the corresponding current–voltage (I–V) relationships for these compounds at concentrations of 0.1, 0.3, 1, 3, and 10 μM, across a voltage range from −60 mV to +60 mV. Average current values (n = 4) and their standard deviations (SD) are plotted, with dashed lines representing baseline (control) current levels in the absence of compounds. The ↕ symbol denotes representative amplitudes of current observed in the presence of each compound. All experiments were repeated four times. Statistical significance was evaluated using one-way ANOVA, with values of *p* < 0.001 (***) considered highly significant.

To evaluate the electrophysiological activity of SAL derivatives, we conducted black lipid membrane (BLM) experiments under a 50/150 mM KCl (cis/trans) gradient (Figure 7). Based on prior activity assessment using oxygraph measurements, two selected conjugates (**1d** and **1f**) were chosen for analysis of their ion transport capabilities. These experiments did not reveal the formation of defined channel-like structures. Instead, we observed irregular disruptions in membrane permeability and occasional membrane rupture events. Both examined compounds altered ionic current flowing through artificial membrane to a level of −10 pA at the potential of −60 mV. These experiments proved the capability of modified SAL of active ionic transportation through biological membranes.

## 3. Materials and Methods

### 3.1. General Procedures

All reagents were purchased from two sources: Merck (Rahway, NJ, USA) or Trimen Chemicals S.A. (Lodz, Poland) and used without further purification. Reaction mixtures were stirred using Teflon-coated magnetic stir bars and were monitored by thin layer chromatography (TLC) using aluminum-backed plates (Merck 60 F_254_). TLC plates were visualized by UV-light (254 nm), followed by treatment with phosphomolybdic acid (PMA) (5% in absolute ethanol) and gentle heating. Products of the reactions were purified using a CombiFlash^®^ Rf+ Lumen Flash Chromatography System (Teledyne Isco) with integrated ELS and UV detectors. All solvents used in flash chromatography were of HPLC grade (Merck) and were used as received. Solvents were removed using a rotary evaporator.

NMR spectra were recorded on a Varian 400 (^1^H NMR at 400 MHz and ^13^C NMR at 101 MHz) magnetic resonance spectrometer or Bruker AvanceNEO 600 USA, (^1^H NMR at 600 MHz and ^13^C NMR at 151 MHz) magnetic resonance spectrometer ^1^H NMR spectra are reported in chemical shifts downfield from TMS using the respective residual solvent peak as internal standard (CD_3_CN δ 1.94 ppm; C_6_D_6_ δ 7.16 ppm; DMSO-d_6_ δ 2.50 ppm; CD_3_OD δ 3.31 ppm). ^1^H NMR spectra are reported as follows: chemical shift (δ, ppm), multiplicity (s = singlet, d = doublet, t = triplet, dd = doublet of doublets, dt = doublet of triplets, dq = doublet of quartets, ddd = doublet of doublet of doublets, td = triplet of dublets, tt = triplet of triplets, tdt = triplet of doublets of triplets, p = quintet, m = multiplet), coupling constant(s) in Hz, and integration. Significant peaks are reported within the overlapping ~2.00–0.50 ppm region of the ^1^H NMR spectra. ^13^C NMR spectra are reported in chemical shifts downfield from TMS using the respective residual solvent peak as internal standard (CD_3_CN δ 1.32 ppm, 118.26 ppm; C_6_D_6_ δ 128.06 ppm; DMSO-d_6_ δ 39.52 ppm; CD_3_OD δ 49.00 ppm). Line broadening parameters were 0.5 or 1.0 Hz, while the error of chemical shift value was 0.1 ppm.

Electrospray ionization (ESI) high resolution mass spectra were recorded on a QTOF (Impact HD, Bruker Daltonics, Billerica, MA, USA) mass spectrometer in the positive ion detection mode. Electrospray ionization (ESI) measurements with positive mode detection were also performed using PurIonMass Spectrometer Model L. Samples were prepared in dry acetonitrile. The mass range for ESI experiments was from *m*/*z* = 400 to *m*/*z* = 1450.

### 3.2. Isolation of Salinomycin and Monensin

Salinomycin (SAL) was isolated as sodium salt from commercially available veterinary premix Sacox^®^, using the procedure described previously [54]. The obtained sodium salt of SAL was then extracted with a solution of sulfuric acid (pH = 1), giving oily SAL ready for further synthesis. After the 3-times repeated evaporation with n-pentane, this oil was transformed to white amorphous solid.

Monensin A (MON) was isolated as sodium salt from commercially available veterinary premix Coxidin^®^, using the procedure described previously [55]. The obtained sodium salt of MON was then extracted with a solution of sulfuric acid (pH = 1), giving oily MON ready for further synthesis. After the 3-times repeated evaporation with *n*-pentane, this oil was transformed to white amorphous solid.

### 3.3. Synthesis of (3-Carboxypropyl)triphenylphosphonium Bromide Propargyl Ester (Compound **3**)

(3-carboxypropyl)triphenylphosphonium bromide (3.00 g, 6.99 mmol, 1.0 equiv.) was dissolved in DMF (30 mL), and the solution was refluxed. After 15 min, DBU (1.81 g, 11.88 mmol, 1.7 equiv.) was added dropwise to the reaction mixture, and after another 20 min, propargyl bromide (2.49 g, 20.96 mmol, 3.0 equiv.) was added dropwise. The resulting solution was stirred and heated for the next 24 h. The reaction mixture was then concentrated on a rotary evaporator. Purification on silica gel using a CombiFlash^®^ system (0% → 10% MeOH/CHCl_3_) gave the pure product as a yellow oil. After twice evaporation to dryness with *n*-pentane, the oily products were completely converted into amorphous solid with a yield of 53% (1.73 g). The product was used straightforwardly in the next step of the study.

### 3.4. Synthesis of (3-Carboxypropyl)triphenylphosphonium Bromide Propargyl Amide (Compound **4**)

(3-carboxypropyl)triphenylphosphonium bromide (3.00 g, 6.99 mmol, 1.0 equiv.) was dissolved in anhydrous DMF (15 mL) and the solution was cooled to −5 °C, upon stirring. Ethyl chloroformate (796 mg, 7.34 mmol, 1.05 equiv.) and triethylamine (1.06 g, 10.48 mmol, 1.5 equiv.) were added dropwise. After 30 min, the solution of propargylamine (346 mg, 6.29 mmol, 0.9 equiv.) in anhydrous DMF was added dropwise. The mixture was allowed to warm to ambient temperature. After 24 h, the reaction mixture was concentrated on a rotary evaporator. The oily residue was dissolved in a small amount of CH_2_Cl_2_ and extracted with brine. Organic phases were concentrated under reduced pressure. Purification on silica gel using a CombiFlash^®^ system (0% → 10% MeOH/CHCl_3_) gave the pure product as a yellow oil. The oil was diluted in *n*-pentane and evaporated to dryness three times to form an amorphous solid, with an isolated yield of 49% (1.59 g). The product was used straightforwardly in the next step of the process.

### 3.5. Synthesis of Triphenylpropargylphosphonium Bromide (Compound **5**)

To a stirred solution of tributylphosphine (5.00 g, 4.94 mmol, 1.0 equiv.) in 1,4-dioxane (10 mL), a 42% aqueous solution of hydrobromic acid (2 mL) was added dropwise at room temperature. The resulting mixture was stirred for additional 50 min at ambient conditions. Subsequently, propargyl bromide (2.94 g, 4.94 mmol, 1.0 equiv.) was added dropwise to the reaction mixture. The reaction was allowed to proceed for 72 h at room temperature. After completion, the mixture was concentrated under reduced pressure, and the crude residue was used directly in the subsequent step of the study without further purification.

### 3.6. Synthesis of C20-Azide of Salinomycin

The procedure was reproduced based on previously reported data [32]. To a stirred solution of SAL (1.0 equiv.) in dichloromethane, DMAP (5.0 equiv.), TMSEtOH (6.0 equiv.) and TCFH (1.2 equiv.) were successively added. The reaction mixture was cooled in an ice bath for the first 2 h, then the reaction was conducted for 22 h at room temperature. The mixture was concentrated under reduced pressure with silica gel and the residue was purified by flash column chromatography using a CombiFlash^®^ system (0% → 30% hexane/ethyl acetate) giving the C1-silyl ester of SAL as a clear oil. After twice evaporation to dryness with *n*-pentane, the oily products were fully converted into white amorphous solid with a yield of 58%.

To a stirred solution of C1-silyl ester of SAL in anhydrous tetrahydrofuran, triphenylphosphine (1.5 equiv.) was added. After 15 min of stirring, DIAD (1.2 equiv.) was added. After the next 15 min, DPPA (1.1 equiv.) was introduced. The reaction mixture was cooled in an ice bath during the addition of reagents. The mixture was stirred for 3 days and then it was concentrated under reduced pressure to dryness. The residue was purified by flash column chromatography using a CombiFlash^®^ system (0% → 30% hexane/ethyl acetate) giving the C1-silyl ester of C20-*epi*-azido-SAL as a clear oil. After evaporation to dryness with *n*-pentane twice, the oily products were completely converted into white amorphous solid with a yield of 53%.

To a solution of C1-silyl ester of C20-*epi*-azido-SAL in tetrahydrofuran, TBAF (3.0 equiv.) was added. The mixture was stirred for 24 h. Then, the organic solvent was removed by evaporation under reduced pressure to dryness. The residue was purified by flash column chromatography using a CombiFlash^®^ system (0% → 50% hexane/ethyl acetate) giving the C20-epi-azido-SAL as a clear oil. After evaporation to dryness with *n*-pentane twice, the oily products were completely converted into white amorphous solid with a yield of 68%.

### 3.7. General Procedure for Preparation of “Click”-Type Phosphonium Salts of Salinomycin (Analogs **1a**–**1d**)

Under a nitrogen atmosphere, to a solution of C20-azide of SAL (1.0 equiv.) in anhydrous CH_3_CN, a respective propargyl precursor (triphenylpropargylphosphonium bromide, **3**, **4** or **5**) (2.5 equiv.) and DIPEA (3.0 equiv.) were added, followed by the addition of catalytic CuI (0.1 equiv.) in one portion. The reaction mixture was stirred at room temperature for 24 h. After consumption of the substrate (TLC and ESI-MS control), the organic solvent was removed on a rotary evaporator. The oily residue was dissolved in a small amount of CH_2_Cl_2_ and extracted a few times with 10% aq. EDTA solution. Organic phases were concentrated under reduced pressure. Purification on silica gel using a CombiFlash^®^ system (0% → 100% EtOAc/CHCl_3_, then 0% → 30% acetone/CHCl_3_) gave the pure product as a yellow oil. The oil was diluted in *n*-pentane and evaporated to dryness three times to form an amorphous solid.

Phosphonium salt **1a**: 74 mg, 38% yield. Isolated as a white amorphous solid, a single spot by TLC. UV-active and stains green with PMA; ^1^H NMR (400 MHz, CD_3_CN) δ 7.93–7.54 (m, 11H), 6.58–6.39 (m, 2H), 6.08–5.91 (m, 2H), 4.01–3.93 (m, 1H), 3.86 (dd, J = 5.1, 0.8 Hz, 1H), 3.78–3.71 (m, 1H), 3.69–3.58 (m, 3H), 3.49 (dd, J = 9.7, 2.4 Hz, 1H), 3.45–3.32 (m, 2H), 3.12 (ddd, J = 14.8, 7.4, 4.2 Hz, 2H), 3.04–2.91 (m, 2H), 2.68–2.54 (m, 2H), 2.47–2.42 (m, 1H), 2.40 (d, J = 2.4 Hz, 1H), 2.16–2.06 (m, 2H), 2.02–0.06 (m, 53H) ppm; ^13^C NMR (101 MHz, CD_3_CN) δ 214.8, 170.9, 136.1, 136.0, 134.9, 134.8, 134.6, 134.5, 131.7, 131.6, 131.5, 131.3, 108.3, 99.8, 89.7, 79.9, 77.9, 74.5, 74.2, 73.3, 71.5, 70.3, 58.9, 55.7, 49.8, 48.1, 40.7, 39.2, 37.3, 36.9, 33.7, 32.9, 31.9, 30.4, 29.5, 26.8, 25.3, 24.0, 23.9, 22.4, 22.3, 21.6, 20.9, 18.5, 17.6, 16.7, 15.2, 14.8, 13.9, 12.5, 12.1, 8.5, 6.9 ppm (signals overlapped); HRMS (ESI+) *m*/*z*: [M]^+^ Calcd for C_63_H_87_N_3_O_10_P^+^ 1076.6124; found 1076.6120.

Phosphonium salt **1b**: 37 mg, 38% yield. Isolated as a white amorphous solid, a single spot by TLC. UV-active and stains green with PMA; ^1^H NMR (600 MHz, CD_3_CN) δ 7.91–7.67 (m, 16H), 6.59 (dd, J = 10.5, 2.0 Hz, 1H), 6.19 (dd, J = 10.4, 4.2 Hz, 1H), 6.12–6.08 (m, 1H), 5.35 (s, 1H), 5.20–5.10 (m, 3H), 4.01 (dd, J = 10.6, 5.9 Hz, 1H), 3.96–3.85 (m, 2H), 3.71 (dd, J = 13.0, 5.3 Hz, 2H), 3.60 (d, J = 8.5 Hz, 1H), 3.50 (td, J = 13.4, 8.9 Hz, 3H), 3.29 (d, J = 11.1 Hz, 1H), 3.13–2.90 (m, 4H), 2.70 (s, 1H), 2.66–2.59 (m, 4H), 2.48–2.39 (m, 5H), 2.02–0.59 (m, 43H) ppm; ^13^C NMR (151 MHz, CD_3_CN) δ 214.2, 174.9, 172.8, 142.8, 136.1, 136.1, 134.7, 134.6, 131.3, 131.2, 128.5, 127.0, 125.5, 119.4, 118.8, 109.2, 99.8, 89.6, 79.9, 77.7, 75.1, 74.0, 72.7, 71.2, 70.1, 59.9, 58.5, 55.6, 49.4, 47.5, 40.5, 39.0, 37.0, 36.2, 34.4, 34.2, 33.9, 32.2, 31.9, 30.3, 29.0, 27.0, 25.1, 22.9, 22.3, 22.1, 21.8, 20.4, 18.8, 18.3, 17.4, 16.9, 15.2, 14.7, 13.6, 12.3, 11.5, 8.0, 6.9 ppm; (ESI+) *m*/*z*: [M]^+^ Calcd for C_67_H_93_N_3_O_12_P^+^ 1162.6491; found 1163.

Phosphonium salt **1c**: 42 mg, 43% yield. Isolated as a white amorphous solid, a single spot by TLC. UV-active and stains green with PMA; ^1^H NMR (600 MHz, C_6_D_6_) δ 10.09 (d, J = 8.0 Hz, 1H), 8.77 (s, 1H), 7.77–7.62 (m, 6H), 7.49–7.32 (m, 10H), 7.11 (dd, J = 9.8, 1.9 Hz, 1H), 6.11 (d, J = 2.5 Hz, 1H), 5.98 (dd, J = 9.8, 2.9 Hz, 1H), 5.70 (dd, J = 15.3, 8.8 Hz, 1H), 4.66 (dd, J = 23.5, 16.1 Hz, 7H), 4.25 (dd, J = 10.5, 4.9 Hz, 1H), 4.16 (d, J = 9.1 Hz, 1H), 3.78 (d, J = 10.3 Hz, 1H), 3.71–3.62 (m, 1H), 3.45 (d, J = 6.6 Hz, 1H), 3.32–3.25 (m, 1H), 3.19 (s, 1H), 3.01–2.94 (m, 1H), 2.87 (dq, J = 14.5, 7.2 Hz, 1H), 2.73 (d, J = 11.6 Hz, 1H), 2.62–2.53 (m, 1H), 2.49 (d, J = 10.3 Hz, 1H), 2.31–2.22 (m, 1H), 2.11 (s, 1H), 2.05–1.98 (m, 1H), 1.96–1.89 (m, 1H), 1.88–0.54 (m, 49H) ppm; ^13^C NMR (151 MHz, C_6_D_6_) δ 213.3, 180.6, 172.1, 144.6, 134.7, 133.9, 133.9, 130.8, 130.7, 128.8, 125.7, 119.6, 119.1, 112.2, 100.6, 86.3, 77.1, 77.0, 76.7, 72.5, 71.0, 70.9, 67.5, 60.3, 55.1, 50.4, 49.1, 39.1, 38.3, 37.3, 36.4, 33.7, 33.3, 32.9, 31.5, 30.2, 28.8, 27.1, 24.3, 23.8, 23.1, 20.8, 20.4, 19.2, 17.6, 16.6, 15.5, 14.5, 13.7, 13.5, 13.2, 11.6, 7.6, 6.8 ppm; HRMS (ESI+) *m*/*z*: [M]^+^ Calcd for C_67_H_94_N_4_O_11_P^+^ 1161.6651; found 1161.6647, [M+Na]^2+^ Calcd for C_67_H_94_N_4_O_11_PNa^2+^ 592.3272; found 592.3290.

Phosphonium salt **1d**: 52 mg, 40% yield. Isolated as a white amorphous solid, a single spot by TLC. Stains green with PMA; ^1^H NMR (600 MHz, CD_3_CN) δ 6.08–5.88 (m, 1H), 5.69–5.61 (m, 1H), 5.54–5.46 (m, 1H), 4.16 (d, J = 1.7 Hz, 1H), 4.11–4.06 (m, 1H), 4.06–3.95 (m, 2H), 3.93–3.88 (m, 1H), 3.82 (dd, J = 9.8, 5.0 Hz, 1H), 3.75–3.68 (m, 2H), 3.47 (d, J = 11.3 Hz, 1H), 3.21 (ddd, J = 15.4, 10.2, 5.2 Hz, 1H), 3.17–3.10 (m, 1H), 3.05 (ddd, J = 23.5, 13.4, 7.3 Hz, 1H), 2.92 (t, J = 20.5 Hz, 1H), 2.75 (ddd, J = 35.7, 14.1, 7.2 Hz, 2H), 2.50–2.42 (m, 1H), 2.25–2.16 (m, 2H), 2.11–0.59 (m, 78H) ppm; ^13^C NMR (151 MHz, CD_3_CN) δ 215.3, 171.5, 131.6, 128.2, 109.6, 100.3, 87.6, 87.6, 78.0, 75.5, 73.5, 73.4, 72.6, 72.0, 71.4, 71.0, 70.1, 61.3, 57.3, 57.0, 50.3, 48.8, 39.4, 37.2, 37.1, 36.6, 34.5, 33.7, 33.6, 31.7, 30.3, 30.2, 29.2, 29.1, 28.3, 27.9, 26.7, 24.9, 24.8, 24.51, 24.49, 23.83, 23.79, 22.9, 21.82, 21.76, 18.82, 18.72, 16.2, 15.0, 14.9, 14.2, 14.1, 13.9, 13.8, 13.6, 12.3, 11.8, 8.0, 7.8, 6.8 ppm; HRMS (ESI+) *m*/*z*: [M]^+^ Calcd for C_57_H_99_N_3_O_10_P^+^ 1016.7063; found 1016.7029.

### 3.8. Synthesis of C20-Amine of Salinomycin

The procedure was reproduced based on previously reported data with minor modifications [42]. To a stirred solution of C20-azide of SAL in anhydrous CH_2_Cl_2_, triphenylphosphine (3.0 equiv.) was added in one portion, followed by the addition of 0.5 mL of water. The mixture was stirred at room temperature for next 24 h, and the reaction progress was monitored by TLC and ESI-MS. The reaction mixture was then concentrated under reduced pressure. Purification on silica gel using a CombiFlash^®^ system (0% → 50% acetone/CHCl_3_) gave the pure product as a yellow oil. The oil was diluted in *n*-pentane and evaporated to dryness three times to form an amorphous solid with a yield of 42%.

### 3.9. Synthesis of Amide-Type Phosphonium Salt of Salinomycin (Compound **1e**)

(3-carboxypropyl)triphenylphosphonium bromide (250 mg, 0.58 mmol, 1.0 equiv.) was dissolved in anhydrous DMF (15 mL) and the solution was cooled to −5 °C upon stirring. Ethyl chloroformate (57 mg, 0.52 mmol, 0.9 equiv.) and triethylamine (88 mg, 0.87 mmol, 1.5 equiv.) were added dropwise. After 30 min the solution of SAL amine (524 mg, 0.70 mmol, 1.2 equiv.) in anhydrous DMF was added dropwise. The mixture was allowed to warm to ambient temperature. After 24 h, the reaction mixture was concentrated on a rotary evaporator. The oily residue was dissolved in a small amount of CH_2_Cl_2_ and extracted with brine. Organic phases were concentrated under reduced pressure. Purification on silica gel using a CombiFlash^®^ system (0% → 30% MeOH/CHCl_3_) gave the pure product as a yellow oil. The oil was diluted in n-pentane and evaporated to dryness three times to form an amorphous solid, with an isolated yield of 32% (201 mg), a single spot by TLC. UV-active and stains green with PMA; ^1^H NMR (600 MHz, C_6_D_6_) δ 8.57 (s, 1H), 7.80–7.66 (m, 7H), 7.46–7.27 (m, 9H), 6.22 (s, 1H), 5.59 (d, J = 9.7 Hz, 1H), 5.20 (s, 1H), 4.61 (s, 1H), 4.19 (d, J = 37.8 Hz, 3H), 3.97 (s, 3H), 3.49 (d, J = 11.4 Hz, 1H), 3.27 (t, J = 42.7 Hz, 2H), 3.08 (s, 2H), 2.82 (dd, J = 39.6, 34.0 Hz, 3H), 2.47 (s, 1H), 2.26 (s, 1H), 2.04 (d, J = 33.8 Hz, 3H), 1.97–0.63 (m, 51H) ppm; ^13^C NMR (151 MHz, C_6_D_6_) δ 215.8, 178.7, 173.3, 134.6, 134.2, 134.1, 131.0, 130.5, 128.3, 119.4, 118.9, 109.8, 98.7, 86.7, 77.1, 75.4, 74.1, 71.6, 70.8, 69.2, 55.6, 49.9, 49.4, 39.7, 37.6, 35.9, 34.1, 33.8, 31.3, 30.3, 30.2, 28.5, 26.6, 25.7, 23.2, 21.9, 21.5, 21.1, 20.2, 19.5, 19.0, 18.8, 16.4, 14.8, 14.1, 13.7, 12.6, 11.3, 7.7, 7.0 ppm; HRMS (ESI) *m*/*z*: [M]^+^ Calcd for C_64_H_91_NO_11_P^+^ 1080.6324; Found 1080.6338.

### 3.10. Synthesis of Double Phosphonium Salt of Salinomycin (Compound **1f**)

To a stirred solution of SAL (1.8 g, 2.40 mmol, 1.0 equiv.) in pyridine (20 mL) succinic anhydride (954 mg, 9.59 mmol, 4.0 equiv.) was added in one portion and the solution was heated to 60 °C. After 24 h, the reaction mixture was concentrated on a rotary evaporator. The oily residue was dissolved in a small amount of CH_2_Cl_2_ and extracted with a solution of sulfuric acid (pH = 1) and water. Organic phases were concentrated under reduced pressure. Purification on silica gel using a CombiFlash^®^ system (0% → 40% EtOAc/hexane) gave the pure product as a clear oil. The oil was diluted in *n*-pentane and evaporated to dryness three times to form an amorphous solid, with an isolated yield of 30% (600 mg).

This compound (300 mg, 0.35 mmol, 1.0 equiv.) was then dissolved in toluene (15 mL), and the solution was refluxed. After 15 min, DBU (183 mg, 1.20 mmol, 3.4 equiv.) was added dropwise to the reaction mixture, and after another 20 min, 1,6-dibrohexane (516 mg, 2.12 mmol, 6.0 equiv.) was added dropwise. After a few minutes, the color of the solution changed to brown. The resulting solution was stirred for the next 24 h. The reaction mixture was then concentrated on a rotary evaporator. Purification on silica gel using a CombiFlash^®^ system (0% → 30% EtOAc/hexane) gave the pure product as a colorless oil. After twice evaporation to dryness with *n*-pentane, the product remained in an oily form (135 mg, 33% yield).

The obtained dibromide (135 mg, 0.11 mmol, 1.0 equiv.) was then dissolved in acetonitrile (15 mL) and the solution was refluxed. Triphenylphosphine (180 mg, 0.69 mmol, 6.0 equiv.) was added in one portion to the reaction mixture, and the stirring and heating were continued for the next 72 h. The reaction mixture was then concentrated on a rotary evaporator. Purification on silica gel using a CombiFlash^®^ system (0% → 100% MeOH/CHCl_3_) gave the pure product as a colorless oil. The oil was diluted in *n*-pentane and evaporated to dryness three times to form an amorphous solid, with an isolated yield of 34% (55 mg).

Isolated as a white amorphous solid, a single spot by TLC. UV-active and stains green with PMA; ^1^H NMR (600 MHz, DMSO-d_6_) δ 7.94–7.75 (m, 30H), 6.26 (dd, J = 10.9, 2.1 Hz, 1H), 5.78–5.74 (m, 1H), 5.13 (s, 1H), 4.27–4.19 (m, 1H), 4.09–4.03 (m, 1H), 3.98 (t, J = 6.7 Hz, 2H), 3.91–3.84 (m, 3H), 3.66–3.56 (m, 6H), 3.51 (d, J = 10.4 Hz, 2H), 3.29 (d, J = 12.7 Hz, 1H), 3.12 (dd, J = 9.8, 7.2 Hz, 1H), 2.98 (td, J = 10.9, 3.9 Hz, 1H), 2.81 (d, J = 5.5 Hz, 1H), 2.64–2.58 (m, 4H), 2.53 (t, J = 5.4 Hz, 1H), 2.12–0.58 (m, 67H) ppm; ^13^C NMR (151 MHz, DMSO-d_6_) δ 213.5, 175.2, 171.84, 171.77, 134.9, 133.6, 133.5, 133.5, 133.5, 130.2, 130.2, 127.4, 118.8, 118.8, 118.2, 118.2, 103.9, 98.6, 86.4, 79.2, 75.8, 74.1, 71.4, 71.0, 69.5, 68.8, 67.5, 64.1, 63.9, 56.9, 54.9, 47.8, 47.1, 35.9, 34.1, 32.9, 31.3, 29.4, 29.3, 29.2, 28.9, 28.5, 27.8, 27.6, 27.5, 25.6, 24.5, 22.3, 22.1, 21.7, 20.3, 19.9, 19.3, 17.2, 15.3, 14.8, 13.6, 12.5, 11.6, 10.9, 7.1, 6.4 ppm; (ESI+) *m*/*z*: [M]^2+^ Calcd for C_94_H_126_O_14_P_2_^2+^ 770.4312; found 770.

### 3.11. Synthesis of C26-Azide of Monensin

The procedure was reproduced based on previously reported data [37]. To a stirred solution of MON (11.0 g, 16.39 mmol, 1.0 equiv.) in toluene (120 mL), an aqueous solution of 0.1 M Na_2_CO_3_ (150 mL) was added. Subsequently, tosyl chloride (9.37 g, 49.18 mmol, 3.0 equiv.) was introduced in a single portion. The resulting mixture was stirred at ambient temperature for 72 h. Upon completion, the reaction mixture was transferred to a separatory funnel and washed successively with 0.1 M Na_2_CO_3_ and brine. The organic phase was then concentrated under reduced pressure and used directly in the following step.

Assuming full conversion of MON to the corresponding tosylate, sodium azide (3.19 g, 49.09 mmol, 3.0 equiv.) was dissolved in DMSO (30 mL). The crude tosylate, previously dissolved in 20 mL of DMSO, was added to this solution. The reaction mixture was stirred at room temperature for another 72 h. After this period, the mixture was diluted with a large volume of water (over 300 mL) and extracted several times with methylene chloride. Caution: this step poses a risk of explosion due to the potential reaction of sodium azide with chlorinated solvents—careful adherence to the described procedure is essential. Alternatively, the obtained azide may be extracted from the aqueous layer using ethyl acetate. The crude product was purified by silica gel chromatography using a CombiFlash^®^ system (gradient: 0% → 40% EtOAc/hexane), yielding the pure compound as a colorless oil. The oil was diluted in *n*-pentane and evaporated to dryness three times to form an amorphous solid. The isolated yield was 62% (7.10 g).

### 3.12. General Procedure for Preparation of “Click”-Type Phosphonium Salts of Monensin (Analogs **2a** and **2c**)

Under a nitrogen atmosphere, to a solution of C26-azide of MON (1.0 equiv.) in anhydrous CH_3_CN, a respective propargyl precursor (triphenylpropargylphosphonium bromide or **4**) (2.5 equiv.) and DIPEA (3.0 equiv.) were added, followed by the addition of catalytic CuI (0.1 equiv.) in one portion. The reaction mixture was stirred at room temperature for 24 h. After consumption of the substrate (TLC and ESI-MS control), the organic solvent was removed on a rotary evaporator. The oily residue was dissolved in a small amount of CH_2_Cl_2_ and extracted a few times with 10% aq. EDTA solution. Organic phases were concentrated under reduced pressure. Purification on silica gel using a CombiFlash^®^ system (0% → 50% acetone/CHCl_3_) gave the pure product as a yellow or colorless oil. The oil was diluted in *n*-pentane and evaporated to dryness three times to form an amorphous solid.

Phosphonium salt **2a**: 217 mg, 21% yield. Isolated as a white amorphous solid, a single spot by TLC. UV-active and stains green with PMA; ^1^H NMR (600 MHz, DMSO-d_6_) δ 7.90 (tdt, J = 10.0, 5.2, 2.6 Hz, 3H), 7.79–7.73 (m, 16H), 7.00 (dd, J = 15.8, 1.0 Hz, 1H), 5.73 (s, 1H), 4.14–4.08 (m, 1H), 4.04 (d, J = 7.1 Hz, 1H), 3.84 (d, J = 3.9 Hz, 1H), 3.73 (dd, J = 9.5, 1.5 Hz, 1H), 3.68–3.63 (m, 1H), 3.46 (dd, J = 9.7, 4.9 Hz, 1H), 3.18 (s, 3H), 3.03 (d, J = 12.7 Hz, 1H), 2.47 (d, J = 0.7 Hz, 3H), 2.23–1.12 (m, 24H), 0.92–0.90 (m, 3H), 0.89–0.85 (m, 6H), 0.77 (t, J = 6.8 Hz, 6H), 0.71 (d, J = 7.0 Hz, 3H), 0.58 (d, J = 6.9 Hz, 3H) ppm; ^13^C NMR (151 MHz, DMSO-d_6_) δ 171.6, 170.4, 134.9, 133.5, 133.4, 130.3, 130.2, 119.2, 118.6, 106.7, 97.5, 87.2, 85.8, 84.8, 82.9, 79.1, 77.7, 76.7, 69.6, 67.2, 56.9, 55.8, 36.1, 35.6, 35.3, 34.7, 34.6, 34.3, 34.1, 31.1, 30.7, 29.5, 29.0, 28.1, 24.7, 21.7, 21.6, 17.6, 15.9, 15.6, 11.7, 10.8, 9.9, 7.9 ppm; HRMS (ESI+) *m*/*z*: [M]^+^ Calcd for C_57_H_79_N_3_O_10_P^+^ 996.5503; found 996.5496.

Phosphonium salt **2c**: 25 mg, 9% yield. Isolated as a white amorphous solid, a single spot by TLC. UV-active and stains green with PMA; ^1^H NMR (600 MHz, CD_3_CN) δ 7.94–7.67 (m, 16H), 3.53–3.49 (m, 3H), 3.45 (t, J = 5.8 Hz, 3H), 3.39 (ddd, J = 13.5, 10.3, 5.8 Hz, 2H), 3.31 (s, 3H), 3.29–3.26 (m, 3H), 2.88–2.85 (m, 3H), 2.58 (dd, J = 8.4, 4.6 Hz, 2H), 2.42–0.79 (m, 51H) ppm; ^13^C NMR (151 MHz, CD_3_CN) δ 172.5, 171.5, 166.9, 136.1, 136.1, 134.6, 134.5, 131.2, 131.1, 119.3, 118.7, 86.2, 60.8, 58.4, 55.2, 54.8, 49.2, 42.0, 38.4, 35.8, 35.2, 34.4, 34.3, 33.6, 32.7, 32.2, 29.7, 29.5, 27.5, 27.0, 25.8, 24.4, 23.2, 22.8, 22.1, 21.8, 21.0, 20.8, 19.9, 19.6, 18.8, 17.7, 16.5, 14.4, 14.3, 11.6, 8.7 ppm; (ESI+) *m*/*z*: [M]^+^ Calcd for C_61_H_86_N_4_O_11_P^+^ 1081.6031; found 1082.

### 3.13. General Procedure for Preparation of “Click”-Type Phosphonium Salts of Monensin (Analogs **2b** and **2d**)

To a stirred solution of MON azide (1.0 equiv.) and a respective propargyl precursor (**3** or **5**) (2.5 equiv.) in 10 mL of 1:1 MeOH/H_2_O mixture, aq. CuSO_4_ (1 M, 0.5 equiv.) and sodium ascorbate (1.0 equiv.) were added. The reaction mixture was stirred at room temperature for 24 h. After consumption of the substrate (TLC and ESI-MS control), the excess of methanol was removed on rotary evaporator and the aqueous solution was diluted by 10% aq. EDTA solution (10 mL) and extracted twice with CH_2_Cl_2_. Organic phases were concentrated under reduced pressure. Purification on silica gel using a CombiFlash^®^ system (0% → 50% acetone/ CHCl_3_) gave the pure product as a yellow oil. The oil was diluted in *n*-pentane and evaporated to dryness three times to form an amorphous solid.

Phosphonium salt **2b**: 43 mg, 7% yield. Isolated as a white amorphous solid, a single spot by TLC. UV-active and stains green with PMA; ^1^H NMR (600 MHz, CD_3_CN) δ 7.92–7.68 (m, 16H), 5.51 (s, 1H), 4.49 (d, J = 12.5 Hz, 1H), 4.34 (s, 1H), 4.29–4.15 (m, 1H), 3.94 (dd, J = 9.1, 1.9 Hz, 2H), 3.84 (dt, J = 6.7, 3.4 Hz, 1H), 3.78 (s, 1H), 3.74 (d, J = 13.3 Hz, 1H), 3.70–3.62 (m, 1H), 3.52 (dd, J = 11.1, 6.6 Hz, 2H), 3.47 (m, 7H), 3.29–3.24 (m, 7H), 3.23–3.21 (m, 1H), 3.19 (s, 1H), 3.17 (s, 1H), 2.90–2.86 (m, 6H), 2.65–2.59 (m, 3H), 2.41–0.70 (m, 31H) ppm; ^13^C NMR (151 MHz, CD_3_CN) δ 175.7, 172.7, 166.6, 135.8, 134.6, 134.5, 134.5, 134.5, 131.0, 130.9, 119.1, 118.6, 107.9, 87.0, 86.0, 81.6, 69.7, 68.3, 64.1, 63.9, 61.8, 58.3, 55.5, 54.6, 49.0, 41.5, 38.2, 37.5, 36.6, 35.2, 32.4, 32.1, 31.2, 30.3, 29.6, 29.5, 29.5, 29.2, 26.8, 24.2, 22.5, 19.7, 19.4, 17.6, 12.4, 11.1, 10.9, 8.3 ppm; HRMS (ESI+) *m*/*z*: [M]^+^ Calcd for C_61_H_85_N_3_O_12_P^+^ 1082.5871; found 1082.5885.

Phosphonium salt **2d**: 54 mg, 10% yield. Isolated as a white amorphous solid, a single spot by TLC. Stains green with PMA; ^1^H NMR (401 MHz, CD_3_CN) δ 7.90 (s, 1H), 4.70–4.51 (m, 2H), 4.40–4.20 (m, 3H), 4.19–4.04 (m, 2H), 3.99–3.79 (m, 4H), 3.73–3.63 (m, 4H), 3.39–3.36 (m, 3H), 3.34–3.29 (m, 5H), 3.28–3.26 (m, 2H), 3.21 (s, 1H), 2.37–0.75 (m, 64H) ppm; ^13^C NMR (101 MHz, CD_3_CN) δ 176.6, 126.8, 108.5, 97.4, 88.0, 87.5, 87.1, 86.7, 86.1, 84.3, 82.7, 78.9, 77.6, 72.0, 68.4, 58.7, 57.4, 52.5, 48.8, 41.6, 38.0, 37.5, 37.1, 35.8, 32.9, 32.1, 28.6, 27.9, 25.0, 24.9, 24.7, 24.6, 24.6, 24.5, 24.0, 23.9, 19.7, 19.6, 19.2, 19.2, 18.0, 18.0, 17.5, 16.4, 16.3, 16.1, 14.0, 13.6, 13.1, 12.4, 11.4, 8.7 ppm; HRMS (ESI+) *m*/*z*: [M]^+^ Calcd for C_51_H_91_N_3_O_10_P^+^ 936.6437; found 936.6448.

### 3.14. Synthesis of C26-Amine of Monensin

The procedure was reproduced based on previously reported data [37]. Under a nitrogen atmosphere, a stirred solution of compound MON azide (2.0 g, 2.87 mmol, 1.0 equiv.) in methanol was treated with a catalytic amount of palladium on carbon (Pd/C). A hydrogen-filled balloon was then attached to the reaction vessel. The mixture was stirred at room temperature under a hydrogen atmosphere until complete consumption of the azide, as confirmed by TLC and ESI-MS (typically within 72 h, with balloon replacement if needed). Upon completion, the reaction mixture was filtered through a pad of Celite to remove the catalyst and concentrated under reduced pressure. Purification on silica gel using a CombiFlash^®^ system (0% → 30% MeOH/CHCl_3_) gave the pure product as a colorless oil. The oil was diluted in *n*-pentane and evaporated to dryness three times to form an amorphous solid, with an isolated yield of 63% (1.22 g).

### 3.15. Synthesis of Amide-Type Phosphonium Salt of Momensin (Compound **2e**)

The procedure was reproduced based on previously reported data [39]. (3-carboxypropyl)triphenylphosphonium bromide (250 mg, 0.58 mmol, 1.0 equiv.) was dissolved in anhydrous DMF (15 mL) and the solution was cooled to −5 °C upon stirring. Ethyl chloroformate (57 mg, 0.52 mmol, 0.9 equiv.) and triethylamine (88 mg, 0.87 mmol, 1.5 equiv.) were added dropwise. After 30 min, the solution of MON amine (468 mg, 0.70 mmol, 1.2 equiv.) in anhydrous DMF was added dropwise. The mixture was allowed to warm to ambient temperature. After 24 h, the reaction mixture was concentrated on a rotary evaporator. The oily residue was dissolved in a small amount of CH_2_Cl_2_ and extracted with brine. Organic phases were concentrated under reduced pressure. Purification on silica gel using a CombiFlash^®^ system (0% → 30% MeOH/CHCl_3_) gave the pure product as a colorless oil. The oil was diluted in *n*-pentane and evaporated to dryness three times to form an amorphous solid, with an isolated yield of 43% (230 mg), a single spot by TLC. UV-active and stains green with PMA; ^1^H NMR (401 MHz, CD_3_OD) δ 7.93–7.87 (m, 3H), 7.83 (tt, J = 5.4, 1.3 Hz, 4H), 7.81–7.73 (m, 8H), 4.16 (dt, J = 8.7, 6.7 Hz, 1H), 4.10 (dd, J = 8.7, 2.3 Hz, 1H), 3.91 (d, J = 4.6 Hz, 1H), 3.73–3.65 (m, 2H), 3.62–3.59 (m, 1H), 3.54–3.47 (m, 2H), 3.46–3.39 (m, 2H), 3.35 (s, 3H), 3.30 (p, J = 3.3, 1.6 Hz, 2H), 3.22 (d, J = 13.5 Hz, 1H), 2.60–2.53 (m, 3H), 2.28–1.35 (m, 25H), 1.33 (s, 3H), 1.13 (d, J = 7.0 Hz, 3H), 1.03–0.89 (m, 18H) ppm; ^13^C NMR (101 MHz, CD_3_OD) δ 179.1, 174.1, 136.5, 136.4, 134.9, 134.8, 131.7, 131.6, 120.2, 119.3, 108.9, 98.5, 88.9, 87.9, 86.9, 85.0, 83.5, 79.8, 78.1, 72.8, 69.1, 59.0, 42.1, 40.3, 38.6, 38.2, 37.8, 36.8, 36.4, 36.3, 36.2, 36.1, 35.4, 32.9, 32.5, 30.6, 29.2, 26.2, 20.02, 19.99, 18.3, 16.7, 16.5, 12.9, 12.4, 11.5, 8.5 ppm; HRMS (ESI) *m*/*z*: [M]^+^ Calcd for C_58_H_83_NO_11_P^+^ 1000.5698; Found 1000.5732.

### 3.16. Synthesis of Double Phosphonium Salt of Monensin (Compound **2f**)

MON (2.0 g, 2.98 mmol, 1.0 equiv.) was dissolved in DMF (40 mL), and the solution was refluxed. After 15 min, DBU (771 mg, 5.07 mmol, 1.7 equiv.) was added dropwise to the reaction mixture, and after another 20 min, 3-bromopropyltriphenylphosphonium bromide (4.17 g, 8.94 mmol, 3.0 equiv.) was added in one portion. After a few minutes, the color of the solution changed to brown. The resulting solution was stirred for the next 24 h. The reaction mixture was then concentrated on a rotary evaporator. Purification on silica gel using a CombiFlash^®^ system (0% → 40% acetone/CHCl_3_) gave the pure product as a colorless oil. The oil was diluted in *n*-pentane and evaporated to dryness three times to form an amorphous solid (1.9 g, 61% yield).

The obtained solid (1.9 g, 1.80 mmol, 1 equiv.) was then dissolved in pyridine (30 mL) and heated to 60 °C. After that, catalytic amount of DMAP and succinic anhydride (720 mg, 7.20 mmol, 4.0 equiv.) were added. After 24 h, the reaction mixture was concentrated on a rotary evaporator. The oily residue was dissolved in a small amount of CH_2_Cl_2_ and extracted with a solution of sulfuric acid (pH = 1) and water. Organic phases were concentrated under reduced pressure. Purification on silica gel using a CombiFlash^®^ system (0% → 40% MeOH/CHCl_3_) gave the pure product as a clear oil. The oil was diluted in *n*-pentane and evaporated to dryness three times to form an amorphous solid, with an isolated yield of 43% (900 mg).

The obtained solid (450 mg, 0.39 mmol, 1 equiv.) was then dissolved in DMF (20 mL), and the solution was refluxed. After 15 min, DBU (101 mg, 0.66 mmol, 1.7 equiv.) was added dropwise to the reaction mixture, and after another 20 min, 3-bromopropyltriphenylphosphonium bromide (544 mg, 1.17 mmol, 3.0 equiv.) was added in one portion. After a few minutes, the color of the solution changed to brown. The resulting solution was stirred for the next 24 h. The reaction mixture was then concentrated on a rotary evaporator. Purification on silica gel using a CombiFlash^®^ system (0% → 100% MeOH/CHCl_3_) gave the pure product as a colorless oil. The oil was diluted in *n*-pentane and evaporated to dryness three times to form an amorphous solid (96 mg, 16% yield).

Isolated as a white amorphous solid, a single spot by TLC. UV-active and stains green with PMA; ^1^H NMR (401 MHz, DMSO-d_6_) δ 7.93–7.72 (m, 30H), 5.54 (s, 1H), 4.16 (dt, J = 26.0, 6.4 Hz, 5H), 4.02–3.95 (m, 2H), 3.87 (dd, J = 14.5, 5.7 Hz, 3H), 3.74 (d, J = 4.4 Hz, 1H), 3.69–3.59 (m, 5H), 3.54 (dd, J = 8.8, 6.0 Hz, 1H), 3.50–3.44 (m, 1H), 3.41 (t, J = 4.6 Hz, 1H), 3.16 (s, 4H), 2.56 (s, 6H), 2.15–0.67 (m, 46H) ppm; ^13^C NMR (101 MHz, DMSO-d_6_) δ 174.4, 171.7, 171.4, 135.0, 133.6, 133.5, 130.3, 130.2, 118.6, 118.6, 117.7, 117.7, 106.6, 102.4, 95.6, 87.1, 85.8, 84.7, 82.7, 80.9, 78.2, 76.4, 69.8, 67.1, 66.4, 63.5, 63.2, 63.0, 57.7, 36.4, 36.3, 35.9, 35.7, 34.6, 34.3, 34.1, 33.8, 30.9, 30.6, 28.6, 28.5, 24.6, 21.5, 17.7, 17.2, 16.0, 15.6, 12.0, 11.7, 10.8, 7.9 ppm; HRMS (ESI+) *m*/*z*: [M]^2+^ Calcd for C_82_H_106_O_14_P_2_^2+^ 688.3259; found 688.3522.

### 3.17. In Vitro Biological Studies

#### 3.17.1. Culture Cell Lines

The studies were conducted on human cell lines including primary (SW480) and metastatic (SW620) colon, metastatic prostate (PC3), breast (MDA-MB-231), lung (A549) and pancreas (MiaPaCa) cancer obtained from the repository of the Medical University of Warsaw. As non-cancerous cells, human immortal keratinocytes (HaCaT) and Chinese hamster lung fibroblasts (V79) were used. All cell lines were cultured in a recommended medium supplemented with 10% fetal bovine serum, penicillin (100 U/mL) and streptomycin (100 μg/L) at 37 °C and 5% CO_2_ till 80% of confluence and next used for further assays.

#### 3.17.2. MTT Assay

Cell viability was assessed using the MTT assay. Briefly, the cells were seeded in 96-well plates at a density of 5 ×10^3^ cells per well and allowed to adhere overnight. Following treatment with SAL, MON and their derivatives at concentrations ranged 1–40 μM for 72 h, 20 µL of MTT solution (5 mg/mL) was added to each well and the well contents were incubated for 3–4 h at 37 °C. The resulting formazan crystals were dissolved in DMSO: isopropanol (1:1 *v*/*v*), and absorbance was measured at 570 nm using a BioTeK Synergy H1 microplate reader (Winooski, VT, USA). Results were expressed as a percentage of viable cells relative to untreated controls. The IC_50_ values were estimated using GraphPad Prism 8.0.1 software (GraphPad Software). All experiments were performed in at least three independent biological replicates, each consisting of three technical replicates.

#### 3.17.3. Mitochondrial Activity

The cells (5 × 10^4^ per well) were seeded in 12-well plates and treated with SAL, MON and their selected derivatives at respective IC_50_ concentrations. Following a 48 h incubation at 37 °C in a humidified atmosphere containing 5% CO_2_, both adherent and suspended cells were harvested, centrifuged at 800× *g* for 5 min at 4 °C, and washed twice with PBS. Mitochondrial staining was performed using MitoTracker™ Red CMXRos and MitoTracker™ Green FM dyes (Invitrogen, Carlsbad, CA, USA), according to the manufacturer’s instructions. Flow cytometry analysis was conducted using a BD FACS celesta cytometer. MitoTracker Green FM was used to assess mitochondrial mass, while MitoTracker Red CMXRos selectively accumulated in active mitochondria, indicating mitochondrial membrane potential integrity.

#### 3.17.4. Mitochondrial ROS Generation

The cells (5 × 10^4^ per well) were seeded in black 96-well plates and preincubated for 24 h prior to treatment. Following the preincubation, the cells were exposed to SAL, MON, and their respective derivatives at concentrations corresponding to their IC_50_ values. The amount of mitochondrial reactive oxygen species (mtROS) was assessed using a fluorometric Mitochondrial Superoxide Detection Assay (Abcam). The cells were incubated with the test compounds for 6 and 12 h at 37 °C with 5% CO_2_. Subsequently, the cells were stained with MitoROS 580, a cell-permeable dye that selectively reacts with mitochondrial superoxide in live cells, producing a red fluorescence signal. Fluorescence was measured at excitation/emission wavelengths of 540/590 nm using a BioTek Synergy H1 microplate reader.

### 3.18. Biophysical Studies

#### 3.18.1. Cell Culture

Oxygraph-2K experiments were conducted using the human alveolar basal epithelial adenocarcinoma cell line A549 (Merck, Darmstadt, Germany). The cells were cultured in T75 flasks (Googlab™) containing Dulbecco’s Modified Eagle Medium (DMEM; Merck, Darmstadt, Germany) supplemented with 10% fetal bovine serum (FBS; Gibco, Thermo Fisher Scientific, Waltham, MA, USA) and antibiotics (100 U/mL penicillin and 100 µg/mL streptomycin; Merck, Darmstadt, Germany). The cultures were maintained at 37 °C in a humidified atmosphere with 5% CO_2_ and passaged upon reaching approximately 90% confluence.

#### 3.18.2. High-Resolution Respirometry

High-resolution respirometry was conducted using the Oxygraph-2K system (Oroboros Instruments, Innsbruck, Austria) following established protocols. Briefly, A549 cells were harvested, centrifuged, and resuspended in starved DMEM containing 1% FBS at a density of 1 × 10^6^ cells/cm^3^ in 2 mL chambers. The cells were then introduced into the respiratory chambers to measure basal respiration. After the respiration stabilized, derivatives of SAL and MON were added at concentrations of 0.1, 0.3, and 1. Maximal electron transfer system (ETS) capacity was assessed by adding 1 μM FCCP. Results are presented as mean ± standard deviation (SD).

#### 3.18.3. Black Lipid Membrane Technique (BLM)

Asolectin, dissolved in *n*-decane at a final concentration of 25 mg/mL, was used to model a biological membrane. The lipid solution was applied using a polyethylene brush to a 250 μm diameter aperture in a Teflon cup, effectively separating the *cis* and *trans* chambers [56]. To enhance the bilayer stability, the perimeter of the aperture was pre-coated with a lipid solution and dried under nitrogen gas prior to membrane formation. Measurement chambers were filled with 1 mL of electrolyte solution containing 50 mM KCl (*cis*), 150 mM KCl (*trans*), and 10 mM HEPES, adjusted to pH = 7.2. The solutions were continuously stirred using magnetic stirrers. Silver/silver chloride (Ag/AgCl) electrodes, connected via agar salt bridges (3 M KCl) to minimize liquid junction potentials, were inserted into each chamber and interfaced with a BLM-120 amplifier (Bio-Logic, Seyssinet-Pariset, France). The electrical signal was digitized using a PowerLab 2/25 converter (ADInstruments, Sydney, Australia), recorded with LabChart5 software, and analyzed using Clampfit 8. All experiments were conducted at room temperature (25 °C) within a Faraday cage to eliminate external electromagnetic interference. During the actual experiments, derivatives of SAL were added to the trans chamber at final concentrations of 0.1, 0.3, 1, 3, or 10 μM. Bilayer formation and thinning were monitored by measuring membrane capacitance and through visual inspection. Only bilayers with final capacitance values between 110 and 180 pF were included in the analysis. A voltage was applied to the cis chamber, while the trans chamber was grounded. Single-channel current data were low-pass filtered at 500 Hz and digitized at a sampling rate of 100 kHz. Recordings were made at varying voltages, stepped in 20 mV increments. Data are presented as mean ± standard deviation (SD).

## 4. Conclusions

In this study, the synthesis of ionophore antibiotic conjugates, salinomycin (SAL) and monensin (MON), with phosphonium cations was performed. Despite the structural similarity of the two starting substrates, different synthetic approaches were required. Distinct linkers were employed, including ester and amide bonds, as well as a 1,2,3-triazole ring, formed via a “click” reaction. Depending on the reagents used, synthetic conditions, and starting material, the desired compounds were obtained with the yields of 7–43%. In terms of antiproliferative activity, several compounds exhibited lower IC_50_ values against cancer cell lines compared to the parent molecules. Notable examples include doubly modified ionophore derivatives (compounds **1f** and **2f**) and selected MON “click” derivatives (compounds **2a** and **2b**). Importantly, nearly all compounds demonstrated the cytotoxicity toward the non-tumorigenic HaCaT cell line lower than the reference drug doxorubicin, while some conjugates were even more potent than the standard treatment. Comparison of these data with previously reported IC_50_ values for ester derivatives at the C1 position of both ionophores indicates that, from a medicinal chemistry perspective, conjugation of the SAL molecule with the TPP^+^ moiety at the C1 carboxyl group is more favorable than modification at the C20 hydroxyl group. In contrast, MON can be efficiently bioconjugated with the TPP^+^ moiety at either the C1 or C26 positions, yielding highly bioactive conjugates. Regarding mitochondrial activity, MitoTracker assays revealed an increase in mitochondrial mass without corresponding changes in mitochondrial membrane potential (ΔΨm). This dissociation between mitochondrial content and membrane polarization suggests that the tested compounds may induce mitochondrial biogenesis or fission independently of the functional activation of the organelle. Furthermore, studies on mtROS generation indicate distinct mechanisms of ion transport and mitochondrial targeting between SAL and MON derivatives. SAL conjugates induced mtROS production in both tested cancer cell lines, an effect not observed for MON derivatives (except for compound **2a**). The synthesized compounds were also examined using biophysical methods. Black lipid membrane (BLM) experiments demonstrated that SAL conjugates retained their ion-transporting capabilities across biological membranes, which could locally alter ion concentrations and, consequently, affect cellular metabolism. Observations such as increased or decreased oxygen flux upon direct compound application, as well as no response to FCCP treatment (used as an uncoupler), support the conclusion that the tested compounds interact directly and immediately with mitochondria.

## Figures and Tables

**Table 1:** Antiproliferative activity (IC_50_, µM) of salinomycin (SAL), monensin (MON) and its derivatives estimated by the MTT assay.

Compound	Cancer Cells						Normal Cells	
	SW480	SW620	PC3	MDA-MB-231	A549	MiaPaCa	HaCaT	V79
	IC_50_	IC_50_	IC_50_	IC_50_	IC_50_	IC_50_	IC_50_	IC_50_
SAL, **1**	1.1 ± 0.04	1.5 ± 0.06	2.7 ± 0.1	1.0 ± 0.1	1.6 ± 0.1	2.6 ± 0.1	0.6 ± 0.1	1.2 ± 0.1
**1a**	9.0 ± 3.6	16.4 ± 2.4	1.2 ± 0.2	3.4 ± 0.5	2.9 ± 0.5	7.6 ± 1.3	3.8 ± 0.3	4.8 ± 0.6
**1b**	8.8 ± 2.4	16.8 ± 4.4	5.5 ± 1.4	5.2 ± 0.2	3.9 ± 0.8	8.5 ± 1.5	2.8 ± 0.2	4.5 ± 0.4
**1c**	9.8 ± 2.0	14.0 ± 4.1	12.9 ± 1.3	11.4 ± 1.3	15.6 ± 9.1	14.1 ± 1.5	7.1 ± 1.2	17.0 ± 2.8
**1d**	2.5 ± 0.6	1.8 ± 0.2	3.6 ± 1.6	5.0 ± 0.3	3.6 ± 0.5	2.9 ± 0.6	2.4 ± 0.1	4.8 ± 0.2
**1e**	10.4 ± 2.3	10.9 ± 2.6	4.0 ± 0.7	6.7 ± 1.2	5.1 ± 0.8	6.4 ± 0.2	8.3 ± 0.7	8.8 ± 0.4
**1f**	0.7 ± 0.2	0.6 ± 0.05	0.3 ± 0.05	1.7 ± 0.002	1.7 ± 0.01	0.4 ± 0.06	1.7 ± 0.002	1.7 ± 0.02
MON, **2**	2.5 ± 0.2	2.7 ± 0.1	3.4 ± 1.0	4.9 ± 0.3	2.6 ± 1.0	1.1 ± 0.2	3.5 ± 1.4	3.6 ± 0.5
**2a**	0.5 ± 0.1	0.7 ± 0.2	0.4 ± 0.1	0.7 ± 0.2	0.3 ± 0.02	0.3 ± 0.1	0.6 ± 0.2	5.0 ± 0.04
**2b**	0.8 ± 0.04	1.5 ± 0.2	0.7 ± 0.3	1.7 ± 0.1	3.4 ± 1.1	1.1 ± 0.3	1.1 ± 0.5	6.9 ± 1.9
**2c**	18.3 ± 0.4	10.6 ± 0.4	14.5 ± 2.1	13.9 ± 2.6	14.9 ± 3.4	13.9 ± 0.7	14.5 ± 3.3	32.2 ± 1.0
**2d**	3.5 ± 0.1	4.3 ± 0.01	3.1 ± 0.6	1.4 ± 0.2	1.4 ± 0.4	1.6 ± 0.3	1.6 ± 0.2	4.2 ± 1.4
**2e**	46.7 ± 3.1	28.1 ± 2.1	>100	>100	>100	>100	39.2 ± 5.6	>100
**2f**	1.7 ± 0.3	2.3 ± 0.4	1.4 ± 0.1	1.4 ± 0.2	1.3 ± 0.2	0.6 ± 0.2	0.3 ± 0.1	3.2 ± 1.1
DOX	0.3 ± 0.08	0.3 ± 0.08	0.6 ± 0.02	0.8 ± 0.03	0.6 ± 0.2	1.1 ± 0.1	0.3 ± 0.01	2.0 ± 0.03

Data are expressed as mean ± SD from at least three independent biological replicates, each consisting of three technical replicates, IC_50_ (µM)—the half-maximal growth inhibitory concentration after cultured the cells for 72 h with the individual compound. Human primary colon cancer (SW480); human metastatic colon cancer (SW620); human metastatic prostate cancer (PC3); human breast cancer (MDA-MB-231); human lung cancer (A549); human pancreas cancer (MiaPaCa); human immortal keratinocyte (HaCaT); Chinese hamster lung fibroblasts (V79). Doxorubicin (DOX)—the selected reference compound commonly used in cancer treatment.

**Table 2:** The calculated values of the selectivity indices (SI) for HaCaT (left side) and V79 (right side) non-tumor cell lines.

Compound		HaCaT					V79					
	SW480	SW620	PC3	MDA-MB-231	A549	MiaPaCa	SW480	SW620	PC3	MDA-MB-231	A549	MiaPaCa
SAL, **1**	0.5	0.4	0.2	0.6	0.4	0.2	1.1	0.8	0.4	1.2	0.8	0.5
**1a**	0.4	0.2	3.2	1.1	1.3	0.5	0.5	0.3	4	1.4	1.7	0.6
**1b**	0.3	0.2	0.5	0.5	0.7	0.3	0.5	0.3	0.8	0.9	1.2	0.5
**1c**	0.7	0.5	0.6	0.6	0.5	0.5	1.7	1.2	1.3	1.5	1.1	1.2
**1d**	1.0	1.3	0.7	0.5	0.7	0.8	1.9	2.7	1.3	1.0	1.3	1.7
**1e**	0.8	0.8	2.1	1.2	1.6	1.3	0.8	0.8	2.2	1.3	1.7	1.4
**1f**	2.4	2.8	5.7	1.0	1.0	4.3	2.4	2.8	5.7	1.0	1.0	4.3
MON, **2**	1.4	1.3	1.0	0.7	1.3	3.2	1.4	1.3	1.1	0.7	1.4	3.3
**2a**	1.2	0.9	1.5	0.9	2.0	2.0	10.0	7.1	12.5	7.1	16.7	16.7
**2b**	1.4	0.7	1.6	0.6	0.3	1.0	8.6	4.6	9.9	4.1	2.0	6.3
**2c**	0.8	1.4	1.0	1.0	1.0	1.0	1.8	3.0	2.2	2.3	2.2	2.3
**2d**	0.5	0.4	0.5	1.1	1.1	1.0	1.2	1.0	1.4	3.0	3.0	2.6
**2e**	0.8	1.4	-	-	-	-	-	-	-	-	-	-
**2f**	0.2	0.1	0.2	0.2	0.2	0.5	1.9	1.4	2.3	2.3	2.5	5.3
DOX	1.0	1.0	0.5	0.4	0.5	0.3	6.7	6.7	3.3	2.5	3.3	1.8

The SI (selectivity index) was calculated using the formula: SI = IC_50_ for normal cell line/IC_50_ for cancer cell line. Human primary colon cancer cells (SW480); human metastatic colon cancer cells (SW620); human metastatic prostate cancers (PC3); human breast cancer cells (MDA-MB-231); human lung cancer cells (A549); human pancreas cancer (MiaPaCa); human immortal keratinocyte cells (HaCaT); Chinese hamster lung fibroblasts (V79). Doxorubicin (DOX)—the selected reference compound commonly used in cancer treatment. SI < 1 high toxicity, SI ≥ 1 low toxicity, SI > 3 highly selective agents.

## Data Availability

The original contributions presented in this study are included in the article. Further inquiries can be directed to the corresponding author.

## References

[1] Liu Y., Sun Y., Guo Y., Shi X., Chen X., Feng W., Wu L.-L., Zhang J., Yu S., Wang Y. (2023). An Overview: The Diversified Role of Mitochondria in Cancer Metabolism. Int. J. Biol. Sci..

[2] Wen S., Zhu D., Huang P. (2013). Targeting Cancer Cell Mitochondria as a Therapeutic Approach. Future Med. Chem..

[3] Battogtokh G., Choi Y.S., Kang D.S., Park S.J., Shim M.S., Huh K.M., Cho Y.-Y., Lee J.Y., Lee H.S., Kang H.C. (2018). Mitochondria-Targeting Drug Conjugates for Cytotoxic, Anti-Oxidizing and Sensing Purposes: Current Strategies and Future Perspectives. Acta Pharm. Sin. B.

[4] Pourahmad J., Salimi A., Seydi E. (2015). Mitochondrial Targeting for Drug Development. Toxicology Studies—Cells, Drugs and Environment.

[5] Global Cancer Observatory. https://gco.iarc.fr/.

[6] Jeena M.T., Kim S., Jin S., Ryu J.-H. (2019). Recent Progress in Mitochondria-Targeted Drug and Drug-Free Agents for Cancer Therapy. Cancers.

[7] Bielski E.R., Zhong Q., Brown M., da Rocha S.R.P. (2015). Effect of the Conjugation Density of Triphenylphosphonium Cation on the Mitochondrial Targeting of Poly(Amidoamine) Dendrimers. Mol. Pharm..

[8] Murphy M.P. (2008). Targeting Lipophilic Cations to Mitochondria. Biochim. Biophys. Acta (BBA)—Bioenerg..

[9] Zielonka J., Joseph J., Sikora A., Hardy M., Ouari O., Vasquez-Vivar J., Cheng G., Lopez M., Kalyanaraman B. (2017). Mitochondria-Targeted Triphenylphosphonium-Based Compounds: Syntheses, Mechanisms of Action, and Therapeutic and Diagnostic Applications. Chem. Rev..

[10] Huczyński A. (2012). Polyether Ionophores—Promising Bioactive Molecules for Cancer Therapy. Bioorg. Med. Chem. Lett..

[11] Antoszczak M., Rutkowski J., Huczyński A. (2014). Structure and Biological Activity of Polyether Ionophores and Their Semisynthetic Derivatives. Bioactive Natural Products.

[12] Gupta P.B., Onder T.T., Jiang G., Tao K., Kuperwasser C., Weinberg R.A., Lander E.S. (2009). Identification of Selective Inhibitors of Cancer Stem Cells by High-Throughput Screening. Cell.

[13] Kopp F., Hermawan A., Oak P.S., Herrmann A., Wagner E., Roidl A. (2014). Salinomycin Treatment Reduces Metastatic Tumor Burden by Hampering Cancer Cell Migration. Mol. Cancer.

[14] Schenk M., Aykut B., Teske C., Giese N.A., Weitz J., Welsch T. (2015). Salinomycin Inhibits Growth of Pancreatic Cancer and Cancer Cell Migration by Disruption of Actin Stress Fiber Integrity. Cancer Lett..

[15] Jangamreddy J.R., Ghavami S., Grabarek J., Kratz G., Wiechec E., Fredriksson B.-A., Rao Pariti R.K., Cieślar-Pobuda A., Panigrahi S., Łos M.J. (2013). Salinomycin Induces Activation of Autophagy, Mitophagy and Affects Mitochondrial Polarity: Differences between Primary and Cancer Cells. Biochim. Biophys. Acta (BBA)—Mol. Cell Res..

[16] Wang F., He L., Dai W.-Q., Xu Y.-P., Wu D., Lin C.-L., Wu S.-M., Cheng P., Zhang Y., Shen M. (2012). Salinomycin Inhibits Proliferation and Induces Apoptosis of Human Hepatocellular Carcinoma Cells In Vitro and In Vivo. PLoS ONE.

[17] Lieke T., Ramackers W., Bergmann S., Klempnauer J., Winkler M., Klose J. (2012). Impact of Salinomycin on Human Cholangiocarcinoma: Induction of Apoptosis and Impairment of Tumor Cell Proliferation in Vitro. BMC Cancer.

[18] Mao J., Fan S., Ma W., Fan P., Wang B., Zhang J., Wang H., Tang B., Zhang Q., Yu X. (2014). Roles of Wnt/β-Catenin Signaling in the Gastric Cancer Stem Cells Proliferation and Salinomycin Treatment. Cell Death Dis..

[19] Lu W., Li Y. (2014). Salinomycin Suppresses LRP6 Expression and Inhibits Both Wnt/Β-Catenin and MTORC1 Signaling in Breast and Prostate Cancer Cells. J. Cell Biochem..

[20] Lu Y., Ma W., Mao J., Yu X., Hou Z., Fan S., Song B., Wang H., Li J., Kang L. (2015). Salinomycin Exerts Anticancer Effects on Human Breast Carcinoma MCF-7 Cancer Stem Cells via Modulation of Hedgehog Signaling. Chem. Biol. Interact..

[21] Riccioni R., Dupuis M.L., Bernabei M., Petrucci E., Pasquini L., Mariani G., Cianfriglia M., Testa U. (2010). The Cancer Stem Cell Selective Inhibitor Salinomycin Is a P-Glycoprotein Inhibitor. Blood Cells Mol. Dis..

[22] Fuchs D., Daniel V., Sadeghi M., Opelz G., Naujokat C. (2010). Salinomycin Overcomes ABC Transporter-Mediated Multidrug and Apoptosis Resistance in Human Leukemia Stem Cell-Like KG-1a Cells. Biochem. Biophys. Res. Commun..

[23] Verdoodt B., Vogt M., Schmitz I., Liffers S.-T., Tannapfel A., Mirmohammadsadegh A. (2012). Salinomycin Induces Autophagy in Colon and Breast Cancer Cells with Concomitant Generation of Reactive Oxygen Species. PLoS ONE.

[24] Ketola K., Hilvo M., Hyötyläinen T., Vuoristo A., Ruskeepää A.-L., Orešič M., Kallioniemi O., Iljin K. (2012). Salinomycin Inhibits Prostate Cancer Growth and Migration via Induction of Oxidative Stress. Br. J. Cancer.

[25] Kim J., Chae M., Kim W.K., Kim Y., Kang H.S., Kim H.S., Yoon S. (2011). Salinomycin Sensitizes Cancer Cells to the Effects of Doxorubicin and Etoposide Treatment by Increasing DNA Damage and Reducing P21 Protein. Br. J. Pharmacol..

[26] Ketola K., Vainio P., Fey V., Kallioniemi O., Iljin K. (2010). Monensin Is a Potent Inducer of Oxidative Stress and Inhibitor of Androgen Signaling Leading to Apoptosis in Prostate Cancer Cells. Mol. Cancer Ther..

[27] Park W.H., Kim E.S., Kim B.K., Lee Y.Y. (2003). Monensin-Mediated Growth Inhibition in NCI-H929 Myeloma Cells via Cell Cycle Arrest and Apoptosis. Int. J. Oncol..

[28] Park W.H., Kim E.S., Jung C.W., Kim B.K., Lee Y.Y. (2003). Monensin-Mediated Growth Inhibition of SNU-C1 Colon Cancer Cells via Cell Cycle Arrest and Apoptosis. Int. J. Oncol..

[29] Park W.H., Seol J.G., Kim E.S., Kang W.K., Im Y.H., Jung C.W., Kim B.K., Lee Y.Y. (2002). Monensin-Mediated Growth Inhibition in Human Lymphoma Cells Through Cell Cycle Arrest and Apoptosis. Br. J. Haematol..

[30] Sulik M., Jędrzejczyk M., Mielczarek-Puta M., Lim G.Y., Podsiad M., Hoser J., Bednarczyk P., Struga M., Huczyński A. (2025). Mitochondria-Targeting Phosphonium Salts of Monensin A as the Source of Novel Antiproliferative Agents. Eur. J. Med. Chem. Rep..

[31] Jędrzejczyk M., Sulik M., Mielczarek-Puta M., Lim G.Y., Podsiad M., Hoser J., Bednarczyk P., Struga M., Huczyński A. (2025). Anticancer Activity of Salinomycin Quaternary Phosphonium Salts. Eur. J. Med. Chem..

[32] Antonenko Y.N., Jędrzejczyk M., Rokitskaya T.I., Khailova L.S., Kotova E.A., Huczyński A. (2022). Rate of Translocation Across Lipid Bilayer of Triphenylphosphonium-Linked Salinomycin Derivatives Contributes Significantly to Their K+/H+ Exchange Activity on Membranes. Bioelectrochemistry.

[33] Urbaniak A., Reed M.R., Heflin B., Gaydos J., Piña-Oviedo S., Jędrzejczyk M., Klejborowska G., Stępczyńska N., Chambers T.C., Tackett A.J. (2022). Anti-Glioblastoma Activity of Monensin and Its Analogs in an Organoid Model of Cancer. Biomed. Pharmacother..

[34] Jędrzejczyk M., Stępczyńska N., Klejborowska G., Podsiad M., Stefańska J., Steverding D., Huczyński A. (2022). Synthesis and Evaluation of Antibacterial and Trypanocidal Activity of Derivatives of Monensin A. Bioorg. Med. Chem. Lett..

[35] Czerwonka D., Mielczarek-Puta M., Antoszczak M., Cioch A., Struga M., Huczyński A. (2021). Evaluation of the Anticancer Activity of Singly and Doubly Modified Analogues of C20-Epi-Salinomycin. Eur. J. Pharmacol..

[36] Czerwonka D., Barcelos Y., Steverding D., Cioch A., Huczyński A., Antoszczak M. (2021). Singly and Doubly Modified Analogues of C20-Epi-Salinomycin: A New Group of Antiparasitic Agents against Trypanosoma Brucei. Eur. J. Med. Chem..

[37] Sulik M., Graniczny R., Janczak J., Kłopotowska D., Wietrzyk J., Huczyński A. (2025). From Pseudocyclic to Macrocyclic Ionophores: Strategies Toward the Synthesis of Cyclic Monensin Derivatives. J. Org. Chem..

[38] Antoszczak M., Gadsby-Davis K., Steverding D., Huczyński A. (2023). Synthesis of Urea and Thiourea Derivatives of C20-Epi-Aminosalinomycin and Their Activity against Trypanosoma Brucei. Eur. J. Med. Chem..

[39] Sulik M., Workneh E.A., Santana S., Teixeira B., Prudêncio M., Janczak J., Huczyński A. (2025). Chemical Modification of Monensin as a Source of Potent Antiplasmodial Agents. Bioorg. Med. Chem..

[40] Antoszczak M., Mielczarek-Puta M., Struga M., Huczynski A. (2025). Urea and Thiourea Derivatives of Salinomycin as Agents Targeting Malignant Colon Cancer Cells. Anticancer Agents Med. Chem..

[41] Sulik M., Maj E., Wietrzyk J., Huczyński A., Antoszczak M. (2020). Synthesis and Anticancer Activity of Dimeric Polyether Ionophores. Biomolecules.

[42] Czerwonka D., Müller S., Cañeque T., Colombeau L., Huczyński A., Antoszczak M., Rodriguez R. (2022). Expeditive Synthesis of Potent C20-Epi-Amino Derivatives of Salinomycin against Cancer Stem-Like Cells. ACS Org. Inorg. Au.

[43] Chan D.C. (2020). Mitochondrial Dynamics and Its Involvement in Disease. Annu. Rev. Pathol. Mech. Dis..

[44] Friedman J.R., Nunnari J. (2014). Mitochondrial Form and Function. Nature.

[45] Youle R.J., van der Bliek A.M. (2012). Mitochondrial Fission, Fusion, and Stress. Science.

[46] Tilokani L., Nagashima S., Paupe V., Prudent J. (2018). Mitochondrial Dynamics: Overview of Molecular Mechanisms. Essays Biochem..

[47] Chen S., Li Q., Shi H., Li F., Duan Y., Guo Q. (2024). New Insights into the Role of Mitochondrial Dynamics in Oxidative Stress-Induced Diseases. Biomed. Pharmacother..

[48] Shadel G.S., Horvath T.L. (2015). Mitochondrial ROS Signaling in Organismal Homeostasis. Cell.

[49] Suski J.M., Lebiedzinska M., Bonora M., Pinton P., Duszynski J., Wieckowski M.R. (2012). Relation Between Mitochondrial Membrane Potential and ROS Formation. Methods. Mol. Biol..

[50] Mitani M., Yamanishi T., Miyazaki Y., Ōtake N. (1976). Salinomycin Effects on Mitochondrial Ion Translocation and Respiration. Antimicrob. Agents Chemother..

[51] Riddell F.G., Hayer M.K. (1985). The Monensin-Mediated Transport of Sodium Ions Through Phospholipid Bilayers Studied by 23Na-NMR Spectroscopy. Biochim. Biophys. Acta (BBA)—Biomembr..

[52] Antonenko Y.N., Rokitskaya T.I., Huczyński A. (2015). Electrogenic and Nonelectrogenic Ion Fluxes Across Lipid and Mitochondrial Membranes Mediated by Monensin and Monensin Ethyl Ester. Biochim. Biophys. Acta (BBA)—Biomembr..

[53] Itoh Y., Law M.J., Sokoloff L. (2000). Effects of the Na+/H+ Exchanger Monensin on Intracellular PH in Astroglia. Brain Res..

[54] Huczyński A., Janczak J., Stefańska J., Antoszczak M., Brzezinski B. (2012). Synthesis and Antimicrobial Activity of Amide Derivatives of Polyether Antibiotic—Salinomycin. Bioorg. Med. Chem. Lett..

[55] Klejborowska G., Jędrzejczyk M., Stępczyńska N., Maj E., Wietrzyk J., Huczyński A. (2019). Antiproliferative Activity of Ester Derivatives of Monensin A at the C-1 and C-26 Positions. Chem. Biol. Drug. Des..

[56] Hoser J., Dabrowska A., Zajac M., Bednarczyk P. (2023). Changes in Ion Transport Across Biological Membranes Exposed to Particulate Matter. Membranes.

